# Research on Bending Wave Regulation Mechanism and Integrated Vibration Attenuation and Energy Harvesting Characteristics of Graded Piezoelectric Metamaterial Beams

**DOI:** 10.3390/ma19214507

**Published:** 2026-10-26

**Authors:** Xingguo Wang, Qiuju Xie

**Affiliations:** 1 College of Intelligence Science and Engineering, Northeast Agricultural University, Harbin 150030, China; wangxingguo2022@163.com; 2 College of Mechanical and Electrical Engineering, Qiqihar University, Qiqihar 161006, China

**Keywords:** metamaterial, vibration attenuation, energy harvesting, low-frequency, broadband

## Abstract

This paper investigates the graded piezoelectric metamaterial beam as the research subject, conducting an in-depth analysis of the bending wave regulation mechanism and its dual-function capabilities for vibration attenuation and energy harvesting. First, a theoretical model of the graded piezoelectric metamaterial beam is established to examine how different grading schemes govern the structural parameters of individual segments within each unit cell. Second, the transfer matrix method is employed to compute the band structures, thereby elucidating the propagation characteristics of bending waves and the underlying energy harvesting mechanisms across various graded configurations. Subsequently, a quantitative evaluation index tailored to the dual-function performance—vibration attenuation and energy harvesting—is proposed to reveal the influence of grading order on frequency-band broadening and overall performance metrics. Finally, the theoretical findings are validated via finite element (FE) simulations. The results demonstrate that the graded piezoelectric metamaterial beam induces a spatial frequency division effect in bending waves and enables localized concentration of wave-field energy. Compared with the non-graded counterpart, the graded configuration simultaneously achieves vibration attenuation and energy harvesting over a low-frequency broadband range. Among these configurations, the fractional-order and first-order graded designs exhibit the most pronounced enhancement of overall dual-function performance.

## 1. Introduction

Vibration and noise suppression, along with self-powering for micro-electromechanical systems (MEMSs), are two critical technological needs in modern industry, attracting extensive attention from both academia and engineering communities. Taking the aerospace field as an example, enhancing aircraft safety, reliability, and passenger comfort has long been a global research focus, with vibration and noise suppression and structural health monitoring being two key technologies. Due to the characteristics of airframe structures, bending waves are most easily excited and serve as the primary energy carriers; thus, vibration propagation in wings and fuselages mainly occurs through bending waves. Driven by the demands for low- to mid-frequency vibration control—particularly below 600 Hz and generally below 1000 Hz—and self-powering for MEMSs, researchers have recently integrated these two functionalities into a unified system, giving rise to the emerging international research frontier of dual-function structures that simultaneously suppress vibrations and harvest energy. This innovation offers novel solutions to challenges in vibration mitigation and power supply for MEMSs in aerospace, marine, nuclear, and other critical industries.

Metamaterials are artificially engineered, periodically structured materials that emerged in the early 21st century and exhibit extraordinary physical properties—such as negative refraction, subwavelength focusing, and anomalous wave manipulation—not observed in naturally occurring substances [1,2,3]. As an interdisciplinary frontier at the confluence of physics, materials science, mechanical engineering, and applied mathematics, metamaterial research has evolved significantly beyond its original scope in electromagnetic wave control. It now encompasses acoustic metamaterials, elastic metamaterials, and piezoelectric-coupled metamaterial systems, with growing applications in vibration isolation, noise attenuation, and vibrational energy harvesting. Recent advances strategically integrate the intrinsic waveband engineering capabilities of metamaterials—including bandgap formation, directional waveguiding, and localized energy confinement—with the electromechanical transduction properties of piezoelectric smart materials. This integration has yielded substantial theoretical insights and experimental advances in vibration suppression, noise attenuation, and high-efficiency vibrational energy harvesting. To enhance practical deployability, a dual-functional paradigm has been proposed internationally that concurrently achieves vibration/noise suppression and energy conversion [4,5,6]. Under this framework, incident mechanical and acoustic energy is selectively attenuated while simultaneously being transduced into usable electrical power, thereby advancing the design of self-powered, intelligent structural systems.

As early as 2009, Liu et al. [7] pioneered the integration of metamaterials with energy harvesting devices to realize a dual-functional design capable of simultaneously suppressing structural vibrations and harvesting vibrational energy. Specifically, they proposed a metamaterial architecture comprising piezoelectric cantilever beams embedded in a honeycomb lattice. Their analysis demonstrated that such metamaterials generate elastic wave bandgaps in the mid-to-low-frequency regime; within these bandgaps, incident vibrational energy is attenuated in the host structure and preferentially localized within resonant subunits. By embedding energy harvesting elements—such as piezoelectric transducers—directly into these localized resonators, the concentrated mechanical energy can be efficiently converted into usable electrical energy. Each resonant unit functions as a mechanical local oscillator, whose dynamic behavior is well approximated by a spring–mass–damper system rigidly coupled to the host structure: at resonance, the oscillator undergoes out-of-phase motion relative to the host, thereby enabling targeted energy transfer from the global structure to the local resonator. Building upon this foundational work, researchers worldwide have systematically extended the concept of dual-functional metamaterials based on mechanical local oscillators, motivated by practical engineering imperatives—including passive vibration mitigation and the development of self-sustaining sensor networks. To date, two principal design paradigms have emerged: linear local oscillators [8,9,10,11,12,13,14,15,16,17] and nonlinear local oscillators [18,19,20,21,22,23,24,25], both of which have substantially advanced the theoretical foundations and design methodologies for multifunctional metamaterial systems.

Most existing vibration attenuation and energy harvesting metamaterials integrate mechanical local oscillators that exploit mechanical resonance to confine vibrational energy spatially. By contrast, piezoelectric local oscillator-based metamaterials employ an electrically tuned resonant circuit—comprising a shunt inductor and the intrinsic capacitance of a piezoelectric bimorph—to achieve precise control of vibrational energy via electromechanically coupled resonance.

Research on single-function vibration control using piezoelectric metamaterials originated in the early 2000s. A seminal contribution was made by Thorp et al. [26] in 2001, who introduced piezoelectric resonant units into one-dimensional rod structures, thereby constructing a periodic piezoelectric array rod. This configuration enabled systematic investigation of longitudinal wave attenuation and elastic energy localization—offering a structural-parameter-free strategy for bandgap tuning. In 2005, the same research group extended this concept to fluid-filled cylindrical shells [27], implementing periodically arranged piezoelectric rings coupled with resonant electrical circuits along the axial direction and analyzing the resulting dispersion characteristics and attenuation behavior of elastic waves. Subsequently, Ruzzene et al. [28] proposed a two-dimensional periodic piezoelectric array plate in 2009 and conducted a comprehensive theoretical analysis of its vibrational modes and wave propagation properties. Experimental validation followed in 2010 [29], wherein the influence of key electrical parameters on vibration suppression efficacy was quantitatively evaluated. Building upon these foundational studies, a substantial body of international research has since emerged, systematically addressing three core themes: (i) computational methodologies for bandgap prediction [30], (ii) physical mechanisms and evolutionary dynamics underlying bandgap formation [31,32,33,34], and (iii) strategies for bandgap broadening and performance optimization [35,36,37]. Collectively, these efforts have yielded a robust theoretical framework and extensive experimental evidence supporting the practical deployment of piezoelectric metamaterials in dedicated vibration control applications. Concurrently, drawing inspiration from the developmental trajectory of mechanical oscillator-based metamaterials—and leveraging the maturing theoretical and experimental foundations of single-function piezoelectric systems—research has increasingly shifted toward integrated dual-functional designs that simultaneously achieve vibration attenuation and piezoelectric energy harvesting.

The most prominent contributions to dual-functional piezoelectric metamaterials—designed concurrently for vibration attenuation and energy harvesting—have been advanced by the Erturk research group at the Georgia Institute of Technology [38,39,40,41]. In 2017, Erturk et al. [38] introduced the first integrated framework for simultaneous vibration attenuation and energy harvesting using piezoelectric metamaterials. They established a modal analysis-based methodology for bandgap prediction and numerical characterization of energy harvesting performance in metamaterial beams. Their findings demonstrated that integrating piezoelectric energy harvesting elements into local resonators enables stable electrical power generation without degrading the host structure’s vibration attenuation capability. In 2018, the same group extended this work by performing a rigorous analytical investigation of electromechanical coupling mechanisms within the unified model [39]. In 2021, Erturk et al. [40] proposed a graded-resonator-type piezoelectric metamaterial beam, wherein spatially varying inductance parameters in shunt circuits enabled programmable graded configurations. Comparative evaluations against uniform counterparts confirmed that tailored grading strategies substantially broaden the operational bandwidth for concurrent vibration attenuation and energy harvesting. Subsequently, in 2022, the group developed a hybrid-resonator design incorporating both mechanical and piezoelectric resonant units, which markedly enhanced vibration attenuation bandwidth, wave attenuation level, harvested power output, and effective frequency range relative to conventional non-graded uniform architectures [41].

Complementing this body of work, the Tol group at the University of Michigan has also pursued dual-functional piezoelectric metamaterials. Tol et al. [42] (2021) reported a piezoelectric metamaterial beam capable of simultaneous elastic wave attenuation and vibrational energy harvesting. Experimental validation revealed an electromechanical conversion efficiency of up to 95% at the local resonance frequency and a sustained efficiency of 51% across the Bragg bandgap—thereby enabling efficient recovery of energy from reflected waves. Building upon these foundational domestic and international advances, our research group has systematically investigated novel piezoelectric metamaterial beam architectures, including stacked [43], asymmetric [44], and graded configurations [45]. Our studies elucidate the underlying coupling mechanisms and distinct dynamic response characteristics associated with each architecture. Collectively, these efforts not only expand the theoretical foundations of integrated dual-function metamaterials but also furnish actionable design principles and quantitative guidance for low- to mid-frequency vibration control and self-powered MEMS.

Although existing research has yielded numerous innovative insights, several fundamental scientific questions in this domain remain unresolved. A critical limitation of piezoelectric local resonant bandgaps—their inherently narrow frequency bandwidth—directly constrains the operational effectiveness of piezoelectric metamaterial beams for simultaneous vibration attenuation and energy harvesting. Consequently, developing robust, scalable strategies to broaden this bandwidth constitutes a pressing priority for advancing practical applications. To date, graded design has emerged as a promising paradigm for widening the effective operating frequency band of such metamaterials. Current approaches fall into two principal categories: geometric grading of piezoelectric elements [46,47] and circuit-parameter grading [40,48]. In the former category, Tang et al. [46] (2022) introduced a graded piezoelectric metamaterial beam incorporating parallel resonant circuits, wherein electrode pairs were spatially arranged with graded dimensions; theoretical analysis demonstrated that, under optimal resistance matching, this configuration achieves the maximal attainable vibration attenuation bandwidth. Subsequently, Tang et al. [47] implemented a geometrically graded layout by longitudinally distributing piezoelectric patches of varying lengths along the beam substrate—yielding a marked expansion of the vibration attenuation band relative to conventional uniform-patch configurations. Both studies exemplify geometric grading strategies. In contrast, circuit-parameter grading—exemplified by Erturk et al. [40]—employs spatially graded impedance shunt circuits across piezoelectric arrays, thereby synthesizing discrete local bandgaps into a contiguous wideband attenuation region. However, this approach entails a practical constraint: low-frequency gradient units necessitate prohibitively large inductance values. To mitigate this issue, Liu et al. [48] substituted the passive LC shunt circuits in [40] with active negative-capacitance circuits, exploiting enhanced electromechanical coupling to realize a continuous attenuation band. Yet, because negative-capacitance circuits rely on active electronic components, their integration compromises the passivity and structural simplicity essential for dual-function systems operating without external power. A comprehensive comparison of the two gradient design strategies reveals that geometric grading of piezoelectric elements offers superior applicability in passive, monolithic dual-function systems.

Inspired by the design concept of broadening the frequency band through geometric grading of piezoelectric elements, this study takes a conventional piezoelectric metamaterial beam as the research foundation and introduces gradient design into the configuration of local resonators. Unlike existing studies on dual-functional gradient piezoelectric metamaterial beams [40,41,45], this work implements gradient control along the axial direction over the geometric symmetry of local resonators without altering external circuit parameters, thereby establishing a theoretical model of a piezoelectric metamaterial beam with spatially varying properties. Based on the precise charge integration method and the transfer matrix method, this study theoretically derives the governing equations of the electric field and structural field, establishes the global transfer matrix that characterizes the bending vibration response, and provides analytical solutions for the corresponding mechanical and electrical quantities during bending wave propagation. Furthermore, this study analyzes the regulation rules of different grading schemes on the structural parameters of each segment within the unit cell and the broadening rules of the dual-functional frequency band and systematically reveals the coupling mechanism and dynamic performance of this new structure for vibration attenuation and energy harvesting. This work aims to achieve the dual-functional output integrating low-frequency operation and broad bandwidth.

## 2. Theoretical Analysis

### 2.1. Model Construction

**Figure 1:**
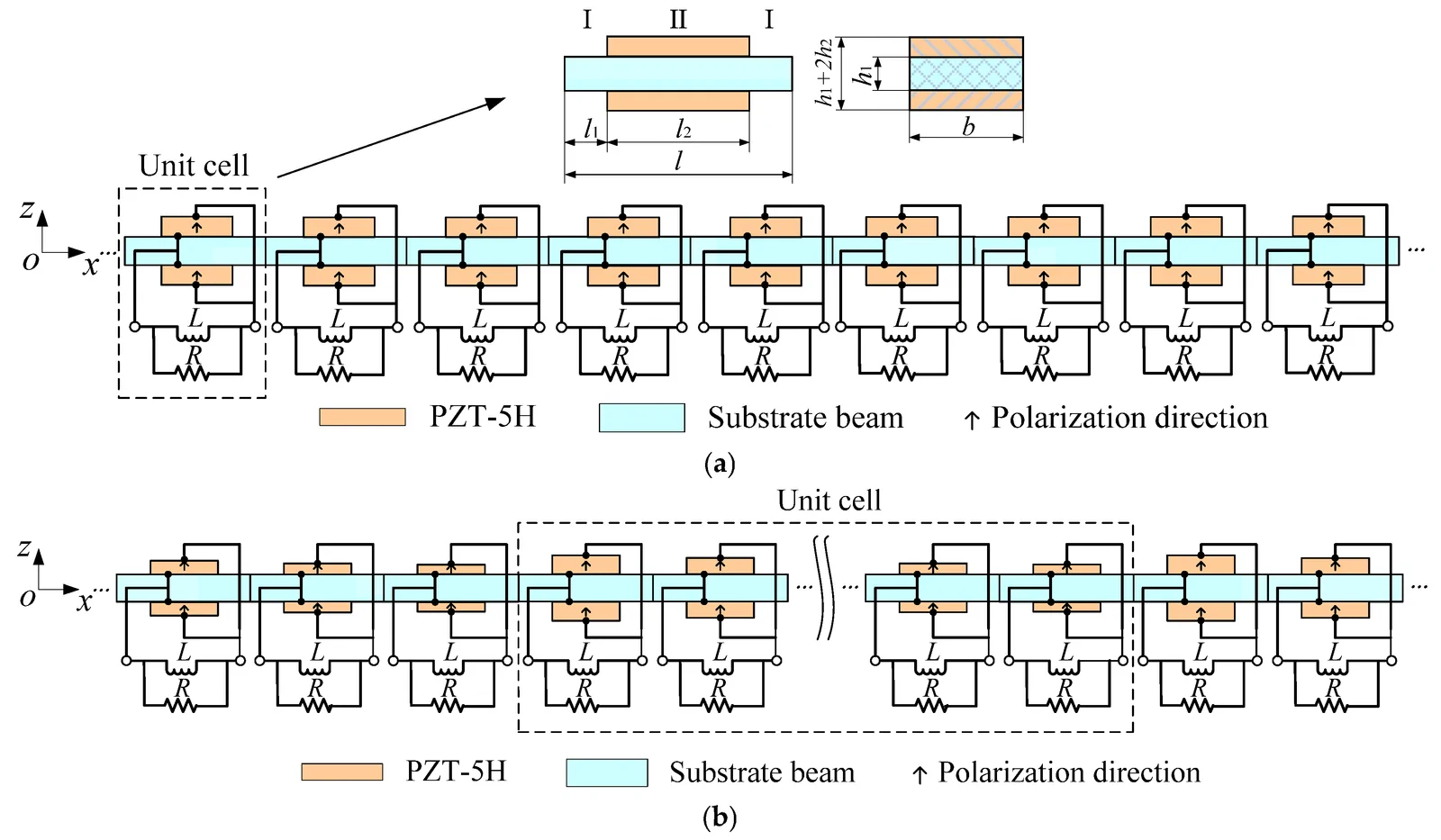
Two-dimensional (2D) schematics of the (**a**) uniform and (**b**) graded metamaterial beams.

This study adopts the conventional uniform piezoelectric metamaterial beam (Figure 1a) as the baseline configuration and proposes a novel integrated beam architecture—capable of simultaneous vibration attenuation and energy harvesting—based on a graded piezoelectric metamaterial design, as illustrated in Figure 1b. The unit cell of the proposed metamaterial beam comprises *S* piezoelectric local oscillators connected in series. To establish a controlled spatial grading in oscillator properties along the beam axis, the first segment retains identical geometric and material parameters as those of the uniform reference structure; in subsequent segments, the thickness of the piezoelectric layer is systematically reduced according to a prescribed power-law grading rule. This tailored gradation effectively broadens the dual-functional operational bandwidth while shifting the effective frequency range toward lower frequencies. Furthermore, to enable systematic parametric evaluation of grading design strategies on dual-functionality, a dimensionless grading order coefficient *p* is introduced as the primary control parameter, following previous studies [40,41,45]. Accordingly, the thickness of the piezoelectric layer in the *n*th segment is analytically expressed as follows:

(1) $$h_{\left(2,n\right)}=h_{2}-\Delta h_{2}{\left(\frac{n-1}{S-1}\right)}^{p}\;n=1,\;2,\;\cdots S$$

where *h*_2_ denotes the initial thickness of the piezoelectric layer in the segmented local oscillator, Δ*h*_2_ represents the allowable variation range of segment thickness, and *S* signifies the total number of segments per unit cell.

**Figure 2:**
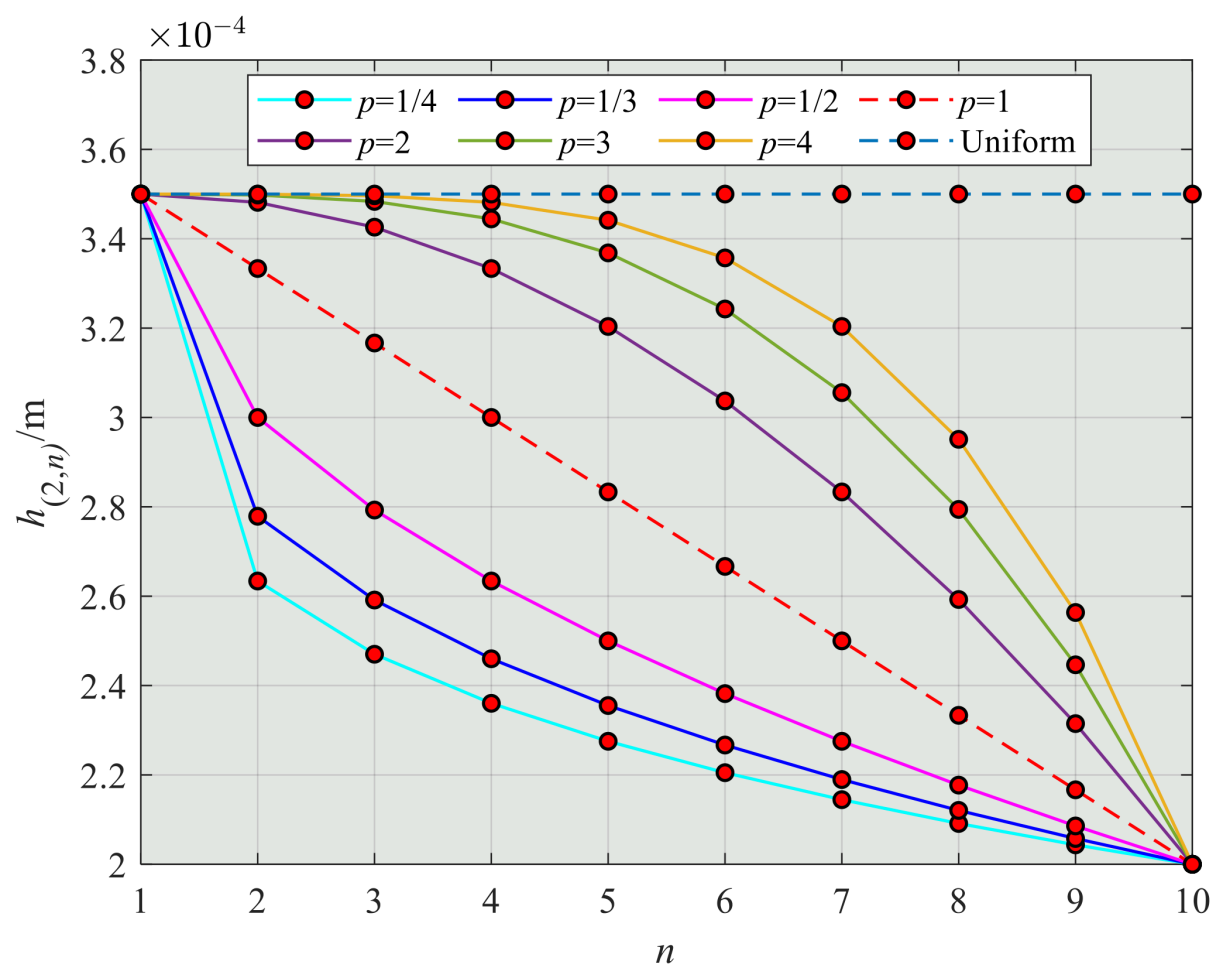
Graded distribution curves of the thickness of each segmented piezoelectric layer in the unit cell across various graded configurations.

This section systematically examines how varying grading orders influence the spatial distribution of structural parameters within the metamaterial beam. Specifically, based on Equation (1), the thicknesses of the piezoelectric layers across individual segmented oscillators are computed, thereby generating grading distribution profiles for piezoelectric layer thickness within each unit cell for various graded configurations—illustrated in Figure 2. The computational setup adopts a reference thickness *h*_2_ = 0.35 mm, a stepwise thickness variation Δ*h*_2_ = 0.15 mm, and a fixed segmentation count *S* = 10. As revealed by the curves in Figure 2, when the total number of segments remains constant, the evolution patterns of piezoelectric layer thickness exhibit marked dependence on the assigned graded order.

When the order *p* = 1, the thickness of the piezoelectric layer in each segment decreases linearly along the beam length, with a constant gradient equal to Δ*h*_2_. The thickness distribution profiles corresponding to fractional-order modes (0 < *p* < 1) and higher-order modes (*p* > 1) are situated on opposite sides of the first-order linear distribution curve. For fractional-order configurations, the rate of thickness reduction progressively diminishes—yielding a pronounced thickness decrease in the leading segments and a comparatively modest reduction in the trailing segments—thereby producing a “front-sparse, rear-dense” spatial distribution along the beam axis. Conversely, for higher-order modes, the thickness decay accelerates monotonically, resulting in a gradual thickness variation near the beam’s leading edge and a sharp decline toward the trailing end—characterizing an overall “front-dense, rear-sparse” distribution pattern.

### 2.2. Construction of the Transfer Matrix

Under the influence of bending waves, the strain at any cross-sectional position *x* within the piezoelectric layer can be expressed as:

(2) $$S(x,t)=\frac{h_{1}+h_{\left(2,n\right)}}{2}\cdot \frac{\partial^{2}w(x,t)}{\partial x^{2}}$$

where *w*(*x*,*t*) = *W*(*x*)e^i*ωt*^ denotes the transverse displacement.

The total charge *Q* generated by the parallel-connected piezoelectric bimorph can be determined via precise charge integration [30], as expressed in the following equation:

(3) $$Q=\frac{d_{31}b(h_{1}+h_{\left(2,n\right)})}{s_{11}^{E}}[\frac{\partial w(l_{2},t)}{\partial x}-\frac{\partial w(0,t)}{\partial x}]+2\varepsilon_{33}^{s}E_{3}A_{s}$$

where $s_{11}^{E}$, *d*_31_, and $\varepsilon_{33}^{s}$ denote the elastic compliance coefficient, the strain coefficient, and the dielectric constant at constant strain of the piezoelectric layer, respectively; *E*_3_ represents the internal electric field strength within the piezoelectric layer; and *A_s_* denotes the effective area of the piezoelectric electrode.

Further mathematical derivation reveals the additional stress *T* induced by the piezoelectric layers located in the upper and lower regions of Zone II, expressed as:

(4) $$T=\frac{\mathrm{sZ}d_{31}^{2}b(h_{1}+h_{\left(2,n\right)})}{{\left(s_{11}^{E}\right)}^{2}h_{1}(1+2\mathrm{sZ}C_{\left(\mathrm{ps},n\right)})}\left[\frac{\partial w(l_{2},t)}{\partial x}-\frac{\partial w(0,t)}{\partial x}\right]$$

where *s* = i*ω* denotes the Laplacian operator, *Z* represents the shunt circuit impedance, and *C*_(*ps*,*n*)_ signifies the intrinsic capacitance of the upper and lower piezoelectric layers within the *n*th segment, given by:

(5) $$C_{\left(\mathrm{ps},n\right)}=\frac{\varepsilon_{33}^{s}A_{s}}{h_{\left(2,n\right)}}$$

This study investigates the bending-dominated vibrational behavior of slender beams, adopting the Euler–Bernoulli beam theory. In this formulation, shear deformation, rotary inertia of the cross-section, and structural damping are explicitly excluded—thereby restricting the model to pure flexural dynamics. The corresponding governing partial differential equation for transverse bending vibration is given by [12]:

(6) $$\frac{\partial^{2}}{\partial x^{2}}\left[E(x)I(x)\frac{\partial^{2}w(x,t)}{\partial x^{2}}\right]+\rho (x)A(x)\frac{\partial^{2}w(x,t)}{\partial t^{2}}=0$$

where *ρ* and *E* denote the material density and Young’s modulus, respectively, and *A* and *I* represent the beam’s cross-sectional area and second moment of area, respectively.

According to Equation (6), analytical expressions for the transverse displacement amplitudes in distinct subregions of a unit cell can be derived independently. Specifically, for Region I—defined as the *n*th non-piezoelectric segment within the *j*th unit cell—the transverse displacement amplitude at an arbitrary cross-section is given by:

(7) $$W_{n,j,1}(x^{\prime })=A_{n,j,1}\cos(\gamma_{1}x^{\prime })+B_{n,j,1}\sin(\gamma_{1}x^{\prime })+C_{n,j,1}\cosh(\gamma_{1}x^{\prime })+D_{n,j,1}\sinh(\gamma_{1}x^{\prime })$$

where $\gamma_{1}=\sqrt[{4}]{\rho_{1}A_{1}\omega^{2}/E_{1}I_{1}}$; *ρ*_1_, *E*_1_, *A*_1_, and *I*_1_ denote the mass density, Young’s modulus, cross-sectional area, and second moment of area of the substrate beam, respectively; *x*^′^ = *x* − *nl*, *nl* ≤ *x* ≤ *nl* + *l*_1_; and *A_n_*_,*j*,1_, *B_n_*_,*j*,1_, *C_n_*_,*j*,1_, and *D_n_*_,*j*,1_ are undetermined coefficients determined by the structural boundary conditions.

In Region II—where the *n*th segmented piezoelectric layer resides within the *j*th unit cell—the transverse displacement amplitude is given by:

(8) $$W_{n,j,2}(x^{\prime \prime })=A_{n,j,2}\cos(\gamma_{2}x^{\prime \prime })+B_{n,j,2}\sin(\gamma_{2}x^{\prime \prime })+C_{n,j,2}\cosh(\gamma_{2}x^{\prime \prime })+D_{n,j,2}\sinh(\gamma_{2}x^{\prime \prime })$$

where $\gamma_{2}=\sqrt[{4}]{(2\rho_{2}A_{2}+\rho_{1}A_{1})\omega^{2}/\left(E_{1}I_{1}+2E_{2}I_{2}\right)}$; *ρ*_2_, *E*_2_, *A*_2_, and *I*_2_ denote the mass density, Young’s modulus, cross-sectional area, and second moment of area of the piezoelectric layer, respectively; *x*^″^ = *x* − (*nl* + *l*_1_), *nl* + *l*_1_ ≤ *x* ≤ *nl* + *l*_1_ + *l*_2_; and *A_n_*_,*j*,2_, *B_n_*_,*j*,2_, *C_n_*_,*j*,2_, and *D_n_*_,*j*,2_ are undetermined coefficients.

Similarly, in Region III—where the *n*th segmented non-piezoelectric layer resides within the *j*th unit cell—the transverse displacement amplitude is expressed as:

(9) $$W_{n,j,3}(x^{\prime \prime \prime })=A_{n,j,3}\cos(\gamma_{1}x^{\prime \prime \prime })+B_{n,j,3}\sin(\gamma_{1}x^{\prime \prime \prime })+C_{n,j,3}\cosh(\gamma_{1}x^{\prime \prime \prime })+D_{n,j,3}\sinh(\gamma_{1}x^{\prime \prime \prime })$$

where *x*^‴^ = *x* − (*nl* + *l*_1_ + *l*_2_), *nl* + *l*_1_ + *l*_2_ ≤ *x* ≤ *nl* + *l*; *A_n_*_,*j*,3_, *B_n_*_,*j*,3_, *C_n_*_,*j*,3_, and *D_n_*_,*j*,3_ are undetermined coefficients.

By differentiating the analytical expressions for displacement amplitudes derived for the aforementioned regions, the amplitude coefficients *θ*(*x*), *M*(*x*), and *Q*(*x*)—representing rotational angle, bending moment, and shear force, respectively—are obtained for each segment.

(10) $$\theta (x)=\left\{\begin{array}{ll} -A_{n,j,1}\gamma_{1}\sin(\gamma_{1}x)+B_{n,j,1}\gamma_{1}\cos(\gamma_{1}x)+C_{n,j,1}\gamma_{1}\sinh(\gamma_{1}x)+D_{n,j,1}\gamma_{1}\cosh(\gamma_{1}x) & x=x^{\prime }\\ -A_{n,j,2}\gamma_{2}\sin(\gamma_{2}x)+B_{n,j,2}\gamma_{2}\cos(\gamma_{2}x)+C_{n,j,2}\gamma_{2}\sinh(\gamma_{2}x)+D_{n,j,2}\gamma_{2}\cosh(\gamma_{2}x) & x=x^{\prime \prime }\\ -A_{n,j,3}\gamma_{1}\sin(\gamma_{1}x)+B_{n,j,3}\gamma_{1}\cos(\gamma_{1}x)+C_{n,j,3}\gamma_{1}\sinh(\gamma_{1}x)+D_{n,j,3}\gamma_{1}\cosh(\gamma_{1}x) & x=x^{\prime \prime \prime } \end{array}\right.$$

(11) $$M(x)=\left\{\begin{array}{ll} E_{1}I_{1}\left[-A_{n,j,1}\gamma_{1}^{2}\cos(\gamma_{1}x)-B_{n,j,1}\gamma_{1}^{2}\sin(\gamma_{1}x)+C_{n,j,1}\gamma_{1}^{2}\cosh(\gamma_{1}x)+D_{n,j,1}\gamma_{1}^{2}\sinh(\gamma_{1}x)\right] & x=x^{\prime }\\ (E_{1}I_{1}+2E_{2}I_{2})\left[-A_{n,j,2}\gamma_{2}^{2}\cos(\gamma_{2}x)-B_{n,j,2}\gamma_{2}^{2}\sin(\gamma_{2}x)+C_{n,j,2}\gamma_{2}^{2}\cosh(\gamma_{2}x)+\right. & \\ \left.D_{n,j,2}\gamma_{2}^{2}\sinh(\gamma_{2}x)\right]+\mathrm{Tb}h_{\left(2,n\right)}(h_{1}+h_{\left(2,n\right)}) & x=x^{\prime \prime }\\ E_{1}I_{1}\left[-A_{n,j,3}\gamma_{1}^{2}\cos(\gamma_{1}x)-B_{n,j,3}\gamma_{1}^{2}\sin(\gamma_{1}x)+C_{n,j,3}\gamma_{1}^{2}\cosh(\gamma_{1}x)+D_{n,j,3}\gamma_{1}^{2}\sinh(\gamma_{1}x)\right] & x=x^{\prime \prime \prime } \end{array}\right.$$

(12) $$Q(x)=\left\{\begin{array}{ll} E_{1}I_{1}\left[A_{n,j,1}\gamma_{1}^{3}\sin(\gamma_{1}x)-B_{n,j,1}\gamma_{1}^{3}\cos(\gamma_{1}x)+C_{n,j,1}\gamma_{1}^{3}\sinh(\gamma_{1}x)+D_{n,j,1}\gamma_{1}^{3}\cosh(\gamma_{1}x)\right] & x=x^{\prime }\\ (E_{1}I_{1}+2E_{2}I_{2})\left[A_{n,j,2}\gamma_{2}^{3}\sin(\gamma_{2}x)-B_{n,j,2}\gamma_{2}^{3}\cos(\gamma_{2}x)+C_{n,j,2}\gamma_{2}^{3}\sinh(\gamma_{2}x)+\right. & \\ \left.D_{n,j,2}\gamma_{2}^{3}\cosh(\gamma_{2}x)\right] & x=x^{\prime \prime }\\ E_{1}I_{1}\left[A_{n,j,3}\gamma_{1}^{3}\sin(\gamma_{1}x)-B_{n,j,3}\gamma_{1}^{3}\cos(\gamma_{1}x)+C_{n,j,3}\gamma_{1}^{3}\sinh(\gamma_{1}x)+D_{n,j,3}\gamma_{1}^{3}\cosh(\gamma_{1}x)\right] & x=x^{\prime \prime \prime } \end{array}\right.$$

At the interface *x* = *nl* + *l*_1_, the boundary compatibility conditions must be rigorously enforced—namely, the displacements, rotations, bending moments, and shear forces across adjacent structural components must be continuous. Based on these conditions, the state transition relationship between Region I and Region II is systematically derived:

(13) $$K_{1,n}\psi_{n,j,2}=H_{1,n}\psi_{n,j,1}$$

where ***K***_1,*n*_ and ***H***_1,*n*_ denote coefficient matrices, $\psi_{n,j,2}={\left[A_{n,j,2}\;B_{n,j,2}\;C_{n,j,2}\;D_{n,j,2}\right]}^{T}$, and $\psi_{n,j,1}={\left[A_{n,j,1}\;B_{n,j,1}\;C_{n,j,1}\;D_{n,j,1}\right]}^{T}$.

Similarly, the state transition relationship between Region II and Region III can be rigorously derived from the continuity condition imposed at *x* = *nl* + *l*_1_ + *l*_2_:

(14) $$K_{2,n}\psi_{n,j,2}=H_{2,n}\psi_{n,j,3}$$

where ***K***_2,*n*_ and ***H***_2,*n*_ denote coefficient matrices; $\psi_{n,j,3}={\left[A_{n,j,3}\;B_{n,j,3}\;C_{n,j,3}\;D_{n,j,3}\right]}^{T}$.

By enforcing continuity at the interface *x* = (*n* − 1)*l*, we derive

(15) $$H_{1,n}\psi_{(n-1),j,3}=H_{2,n}\psi_{n,j,1}$$

where $\psi_{(n-1),j,3}={\left[A_{(n-1),j,3}\;B_{(n-1),j,3}\;C_{(n-1),j,3}\;D_{(n-1),j,3}\right]}^{T}$.

By integrating Equations (13)–(15), the transfer relationship governing the state vectors across adjacent segment end faces is derived.

(16) $$\psi_{n,j,3}=T_{n}\psi_{(n-1),j,3}$$

where ***T****_n_* denotes the transfer matrix, specifically $T_{n}=H_{2,n}^{-1}K_{2,n}K_{1,n}^{-1}H_{1,n}H_{2,n}^{-1}H_{1,n}$.

The metamaterial unit cell comprises *S* graded segments. By sequentially propagating through each segment, the transfer relationship between the corresponding (*S*th) segments of adjacent unit cells can be rigorously established.

(17) $$\psi_{S,j,3}=T_{a}\psi_{S,(j-1),3}$$

where ***T****_a_* denotes the transfer matrix, specifically ***T****_a_* = ***T****_s_****T***_(*s*−1)_ … ***T***_1_.

By incorporating the wave characteristics of periodic structures and applying Bloch’s theorem on periodic boundary conditions, a constraint relationship for the periodic wavevector can be rigorously derived.

(18) $$\psi_{S,j,3}=e^{i\mathrm{kSl}}\psi_{S,(j-1),3}$$

where *k* denotes the Bloch wave vector.

Substituting Equation (18) into Equation (17) yields a standard matrix eigenvalue problem.

(19) $$\left|T_{a}-e^{i\mathrm{kSl}}I\right|=0$$

where ***I*** denotes the 4 × 4 identity matrix.

The dispersion relation between the wave vector *k* and angular frequency *ω* is derived by computing the eigenvalues of the transfer matrix ***T****_a_*, thereby enabling a rigorous characterization of the band structure and vibration bandgap properties of the metamaterial beam.

## 3. Vibration Attenuation Performance Analysis

### 3.1. Calculation and Analysis of the Band Structures

**Figure 3:**
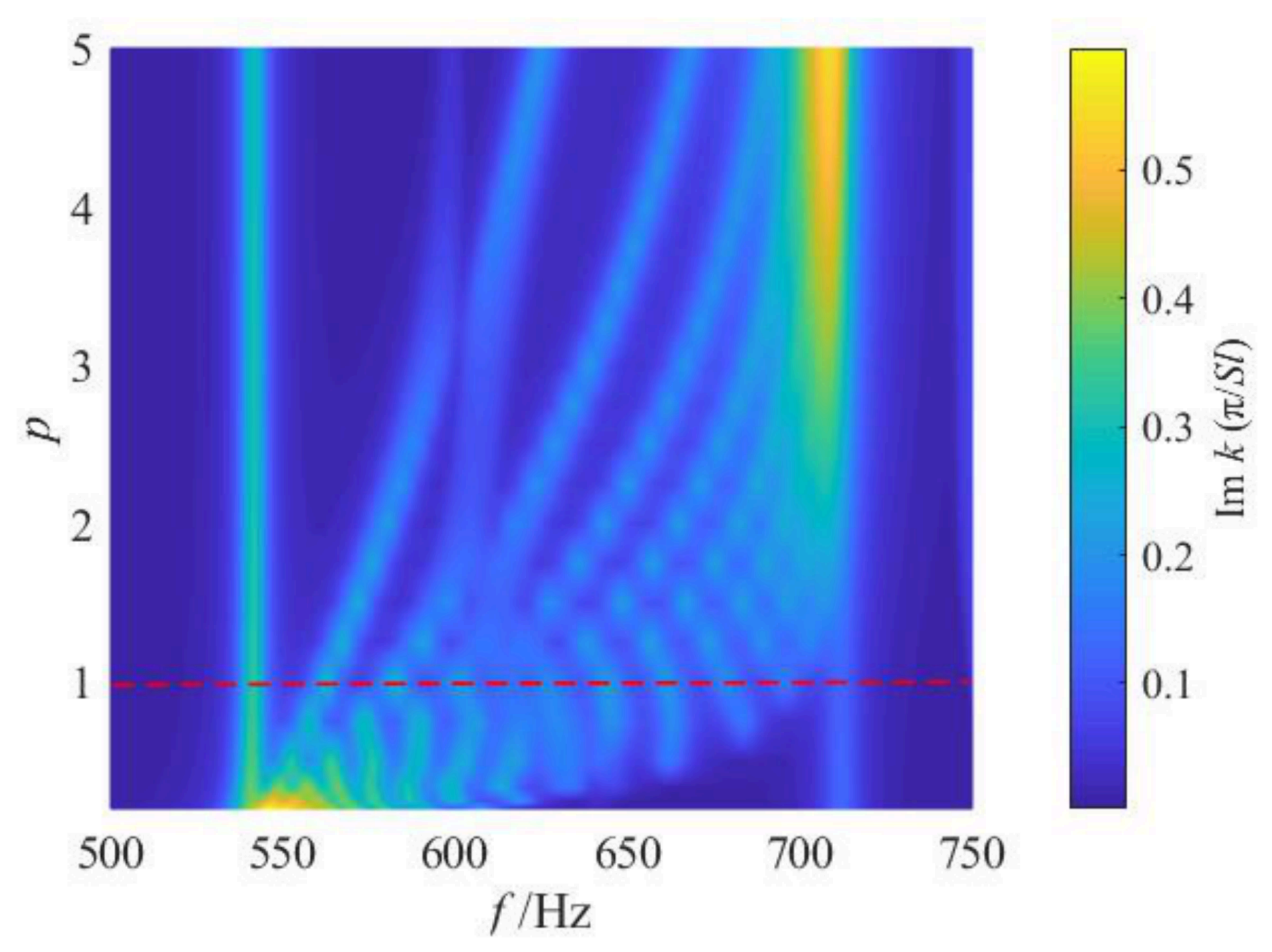
Contour plots illustrating the distribution of bending-wave band structures in graded metamaterial beams across various graded configurations.

To quantitatively characterize the wave propagation characteristics of graded piezoelectric metamaterial beams, the entire structure is modeled as a periodic system comprising identical unit cells. The geometric and material parameters of the unit cell are summarized in Table 1, where the selection of certain geometric, material, and electrical parameters is consistent with that adopted in previous studies [44,45]. Band structures corresponding to various grading orders are computed using Equation (19), with results presented in Figure 3. Analysis of the imaginary part contour plots reveals that bending-wave bandgaps associated with fractional-order grading are predominantly localized in the low-frequency regime. As the grading exponent *p* increases, these bandgaps systematically shift toward higher frequencies; their widths initially increase and subsequently decrease, yielding a characteristic “narrow–wide–narrow” distribution across the frequency spectrum. In contrast, the spatial attenuation constants exhibit an inverse trend—peaking at both spectral extremes and attaining minima near the center.

**Figure 4:**
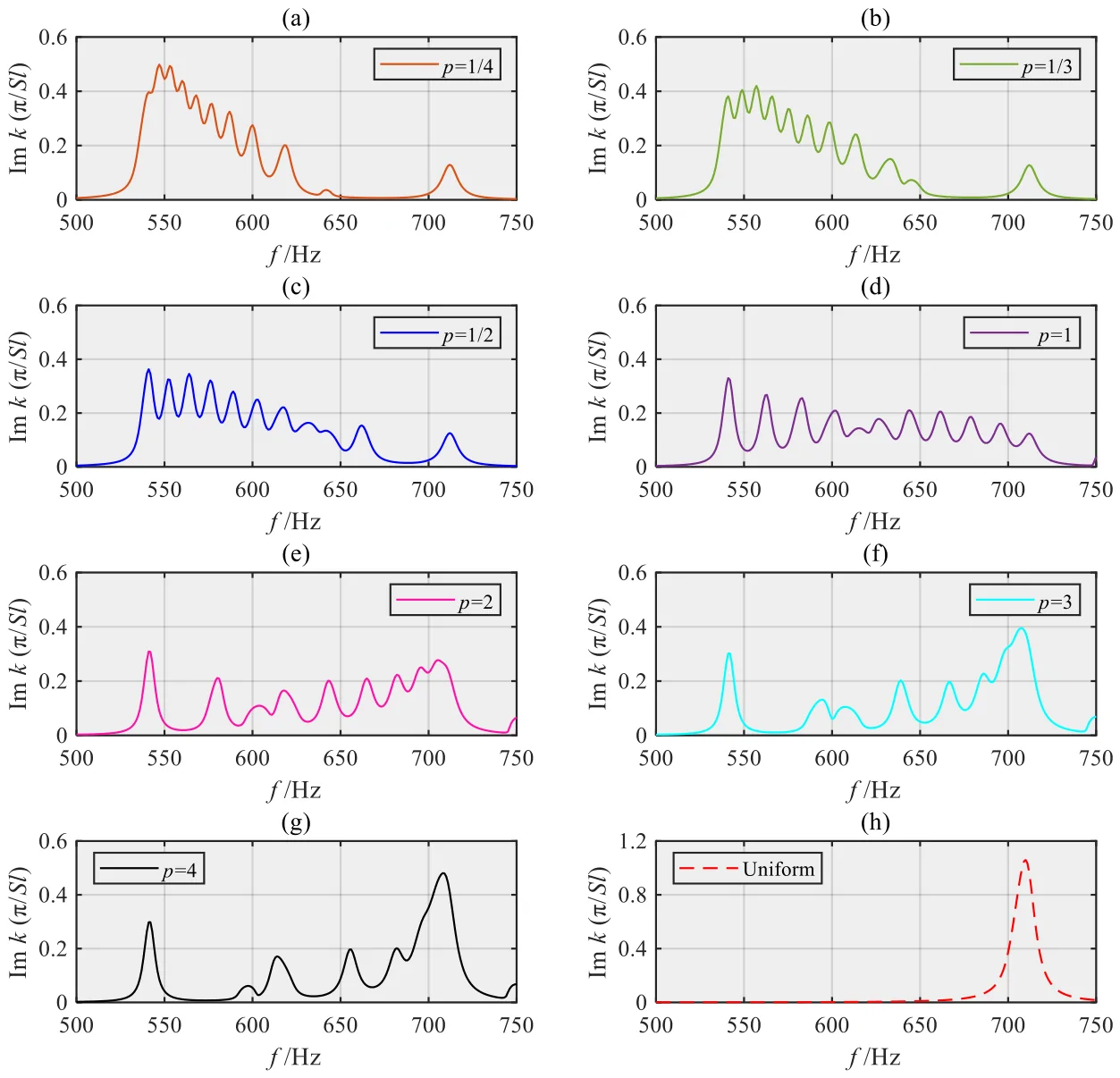
Imaginary part dispersion curves of bending-wave band structures for graded metamaterial beams across various graded configurations.

For direct comparative assessment, Figure 4 presents cross-sectional views of the two-dimensional imaginary part of representative fractional- and integer-order graded configurations, alongside the band structure of a non-graded uniform reference configuration. Comparative analysis indicates that the maximum bandgap width occurs near *p* = 1, whereas the uniform configuration achieves the highest global attenuation constant. To elucidate the physical mechanisms governing this bandgap evolution, the subsequent section conducts a systematic investigation of the intra-cell wave response characteristics, with particular emphasis on the dynamic behavior within each segment of the unit cell.

**Figure 5:**
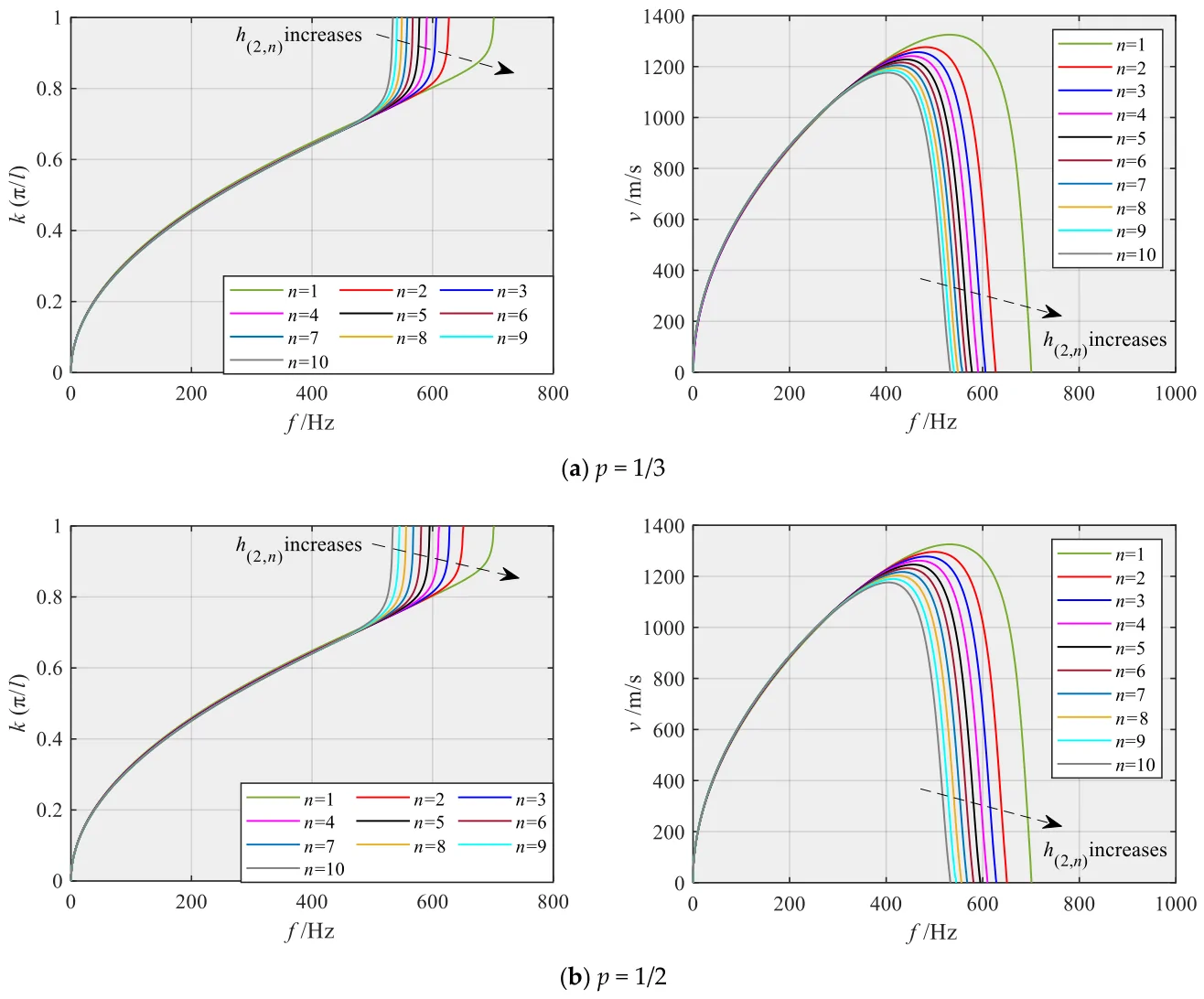

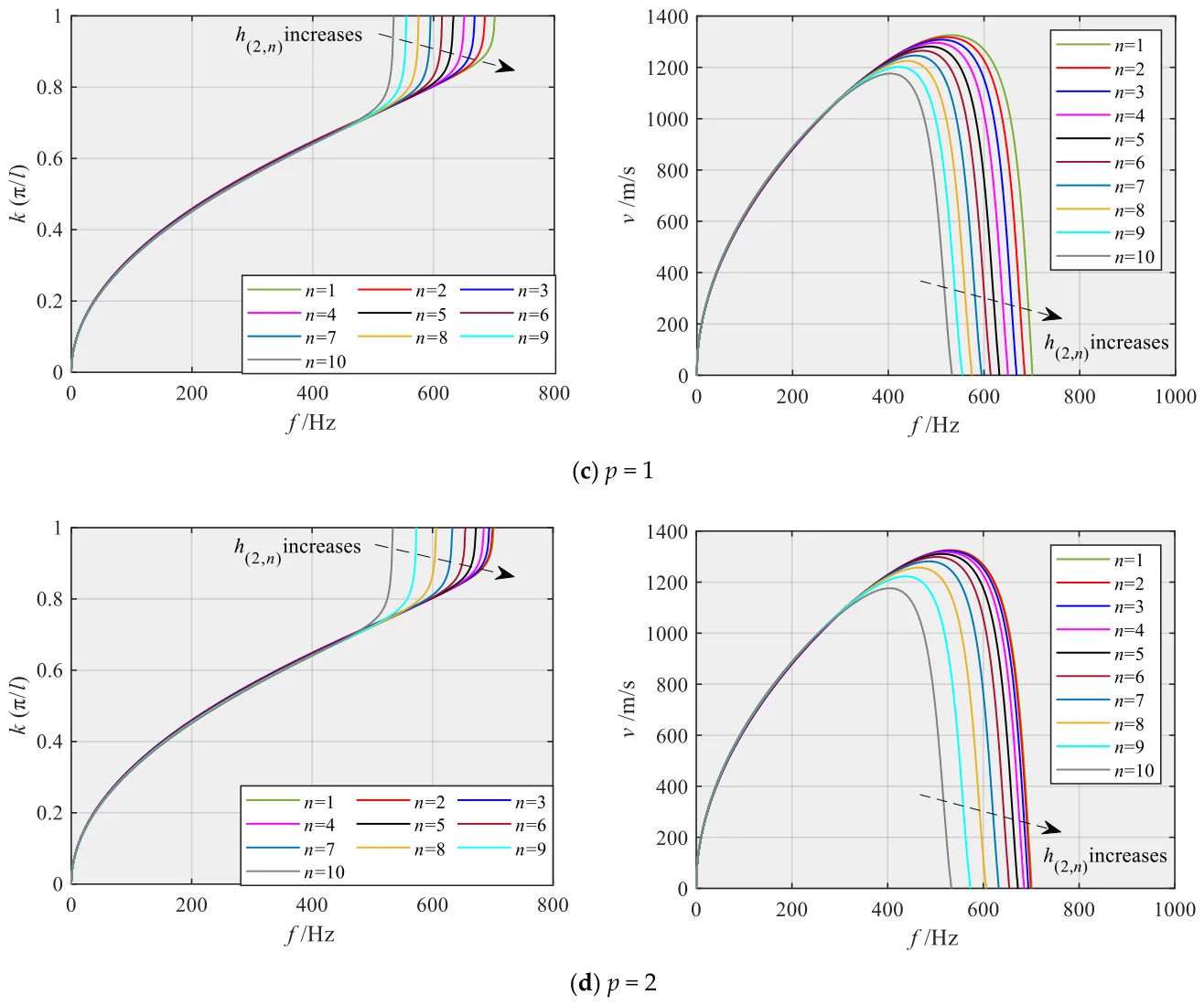
Dispersion curves and corresponding group velocity profiles of bending-wave bands for individual segments within the unit cell across various graded configurations.

Figure 5 presents the real parts of the dispersion relations and corresponding group velocity spectra for all segments across four representative graded unit cell configurations—comprising both fractional-order and integer-order (first- and higher-order) gradients. At low wavevectors, the dispersion branches of individual segments within each configuration exhibit near-coincidence, suggesting minimal sensitivity of wave propagation characteristics to the grading parameter in this regime. With increasing wavevector, the dispersion branches progressively diverge and flatten; upon approaching the Brillouin zone boundary, they fully evolve into dispersionless (flat) bands. Notably, the spatial arrangement of these flat bands—quantified by their longitudinal spacing along the frequency axis—varies systematically with grading order: in fractional-order configurations, the spacing decreases monotonically from higher to lower frequencies; in higher-order configurations, the spacing increases monotonically, whereas in first-order linear graded configurations, the flat bands are uniformly spaced. Complementary analysis of the group velocity spectra reveals that, for each segment, the group velocity rises to a maximum as excitation frequency increases, then declines monotonically, asymptotically approaching zero at frequencies coinciding with the flat-band positions. Moreover, an increase in the thickness *h*_(2,*n*)_ of the segmented piezoelectric layer leads to a proportional upward shift in both the resonant frequencies associated with the flat bands and the zero-crossing frequencies of the group velocity.

By integrating the aforementioned patterns, a frequency-domain wave modulation mechanism is formulated: bending waves of distinct frequencies attain zero group velocity at their respective structural segments, thereby enabling spatial separation between vibration energy localization and electromechanical energy conversion. Specifically, high-frequency bending waves are preferentially coupled with thicker segments, where energy conversion occurs proximal to the excitation source; conversely, low-frequency bending waves couple with thinner segments, shifting the energy conversion regions farther along the propagation direction. Consequently, as bending waves traverse the piezoelectric layer in the direction of decreasing thickness, each frequency component undergoes stopband-induced arrest at its structurally matched segment—yielding a spatially resolved frequency sorting effect, commonly referred to as “rainbow trapping” [49,50,51,52]. From a wavevector perspective, the group velocity vanishes at the Brillouin zone boundaries of individual segments, leading to strong spatial confinement of vibrational energy and consequent amplification of the local wavefield intensity. Crucially, the locally resonant oscillators embedded within each segment of the graded unit cell exhibit segment-specific dynamic responses, collectively accounting for the observed band structure characteristics of the metamaterial beam depicted in Figure 3.

### 3.2. Vibration Transmission Characteristics

An ideal graded piezoelectric metamaterial beam is theoretically modeled as an infinitely periodic structure; however, finite-periodic configurations are employed in practical engineering applications. For such finite-periodic beams, elastic bending waves exhibit pronounced attenuation within the bandgap frequency range. This section conducts numerical calculations in MATLAB R2018b to analyze the vibration transmission response of the finite-length beam, thereby evaluating the vibration suppression performance of the proposed structure. The geometric dimensions, material properties, and shunt circuit parameters adopted across all configurations are summarized in Table 1.

The boundary conditions for the numerical model are specified as follows: transverse harmonic displacement excitation with amplitude *W_in_* is imposed at the left end of the beam. The frequency sweep covers 500 Hz to 750 Hz with a step of 1 Hz, and the acceleration amplitude of excitation remains constant at 10 g; the right end is designated as a free boundary. Under these conditions, the transfer matrix method is employed to compute the transverse displacement amplitude *W_out_* at the right end. Subsequently, the bending vibration transmission ratio is defined, and its analytical expression is provided in Equation (20).

(20) $$\tau (f)=20\mathrm{lg}\left(\frac{W_{\mathrm{out}}}{W_{\mathrm{in}}}\right)$$

**Figure 6:**
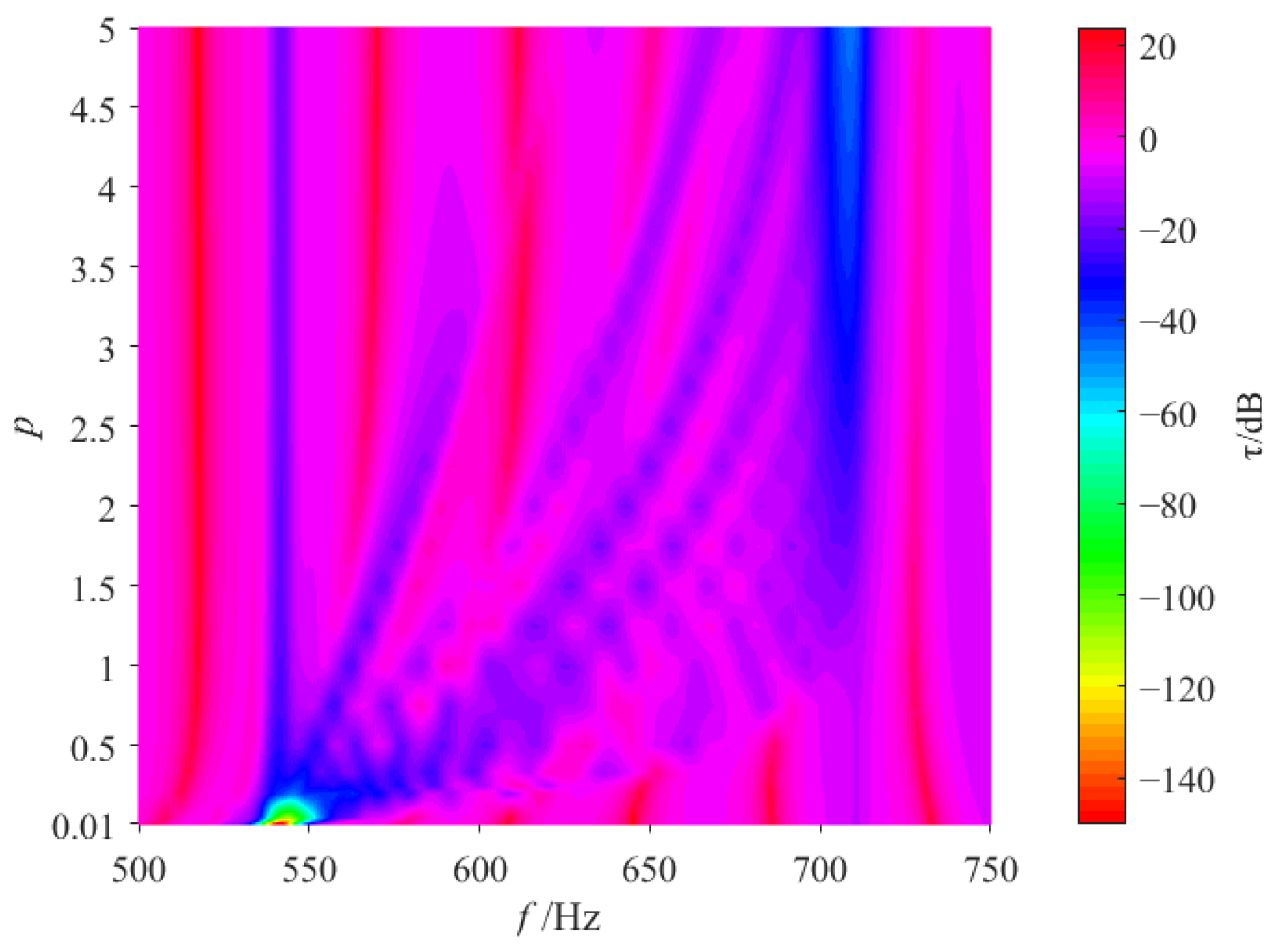
Distribution of vibration transmissibility across various graded configurations.

The vibration transmission characteristics of the structure across various grading orders are numerically investigated using the transfer matrix method, with results presented in Figure 6. Contour plots reveal that the grading order exerts a pronounced influence on both the effective attenuation bandwidth and the peak vibration attenuation amplitude. This behavior is consistent with the bandgap mechanism elucidated earlier and arises from the distinct spatial distributions of resonant frequencies across local oscillators in different structural segments—distributions that are inherently modulated by the specific grading scheme employed.

For the fractional-order grading configuration, the piezoelectric layer thickness in the oscillator decreases more rapidly at the front end, yielding an overall thickness distribution that is relatively sparse near the front and denser toward the rear. When the grading exponent *p* is small, the structure achieves superior low-frequency vibration attenuation; however, its effective attenuation bandwidth remains comparatively narrow. As *p* increases, the thickness difference between adjacent segments at the front end diminishes gradually, promoting overlap and coupling of their individual resonant bandgaps—thereby broadening the overall attenuation bandwidth. Concurrently, the characteristic frequency associated with peak attenuation shifts upward. In contrast, the high-order grading configuration exhibits an inverse thickness evolution: the piezoelectric layer thickness decreases gradually—rather than sharply—at the front end, resulting in an overall distribution that transitions from dense (front) to sparse (rear). With increasing *p*, the front-end thickness discretization becomes finer, reducing inter-segment resonant frequency spacing and enhancing bandgap coupling—leading to significantly improved attenuation performance over the higher-frequency portion of the target range. Conversely, at the rear end, the thickness variation between adjacent segments enlarges, widening the separation between their resonant frequencies and introducing discernible gaps within the attenuation envelope during the lower-frequency portion of the range.

To quantitatively evaluate the impact of grading order on vibration attenuation performance, this section employs four quantitative metrics—attenuation bandwidth (Δ*f*), attenuation area (Φ), maximum attenuation amplitude (*τ*_max_), and the corresponding characteristic frequency (*f*)—to comprehensively characterize the attenuation behavior. The attenuation area Φ is computed according to Equation (21).

(21) $$\Phi =\sum_{i=1}^{t}\int_{f_{si}}^{f_{ci}}\left|\tau (f)\right|\mathrm{df}(\tau (f)<0)t=1,2,3\cdots \;$$

where *t* denotes the total number of segments within the attenuation band, and *f*_s*i*_ and *f*_c*i*_ represent the lower and upper frequency bounds, respectively, of the *i*th attenuation band.

**Figure 7:**
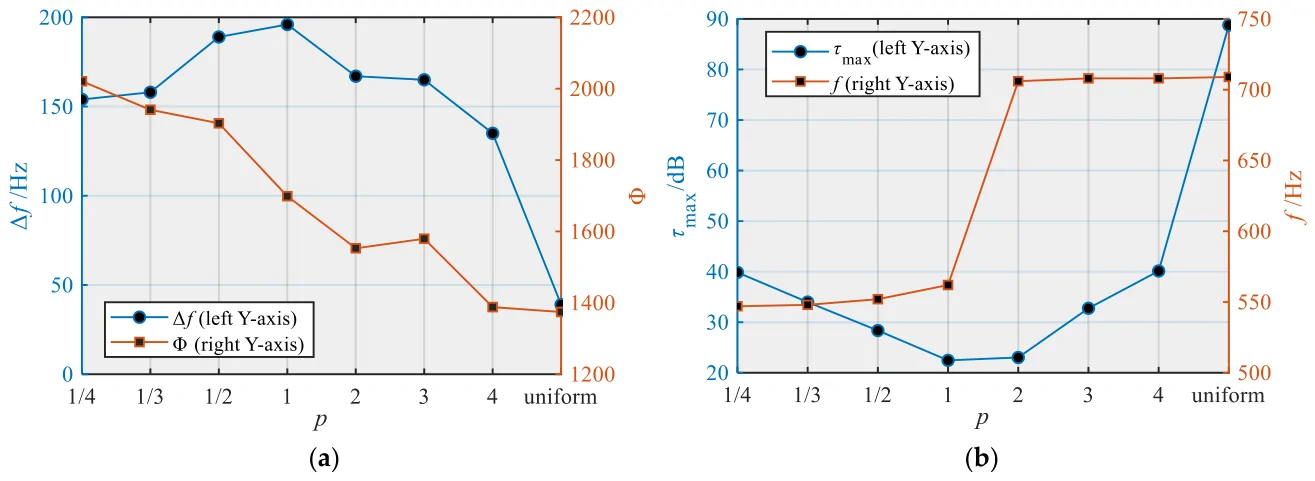
Variation of attenuation bandwidth, attenuation area, maximum attenuation amplitude, and corresponding characteristic frequency across various graded configurations: (**a**) Attenuation bandwidth and attenuation area; (**b**) Maximum attenuation amplitude and its associated characteristic frequency.

Figure 7 presents the numerically computed variation trends of four key vibration attenuation metrics—attenuation bandwidth, attenuation area, maximum attenuation amplitude, and their corresponding characteristic frequencies—across various graded configurations. As shown in Figure 7a, the attenuation bandwidth exhibits a non-monotonic dependence on the grading order *p*: it first increases and then decreases, reaching its global maximum for the first-order graded configuration and its minimum for the non-graded uniform structure. In contrast, the maximum attenuation amplitude *τ*_max_ evolves inversely with bandwidth (Figure 7b): it attains its lowest value at *p* = 1 and peaks for the non-graded case. This trade-off implies that the first-order graded design maximizes frequency coverage for vibration attenuation but sacrifices maximum attenuation performance at individual frequencies; conversely, the uniform configuration delivers superior localized attenuation at discrete frequencies but offers limited broadband effectiveness. Regarding characteristic frequencies, the frequency at which maximum attenuation occurs increases progressively with grading order. Specifically, fractional-order and first-order configurations concentrate maximum attenuation predominantly in the low-frequency regime, whereas higher-order gradients shift the dominant attenuation band toward higher frequencies. Finally, the attenuation area—the integral of attenuation magnitude over frequency—monotonically decreases with increasing grading order. Among all configurations, fractional-order grading yields the largest attenuation area, followed by integer-order gradients; the non-graded uniform structure yields the smallest.

In summary, various graded configuration schemes exhibit distinct trade-offs with respect to attenuation bandwidth, attenuation area, maximum attenuation amplitude, and characteristic frequency. No single configuration simultaneously optimizes all performance metrics. Therefore, in practical engineering applications for vibration mitigation, the selection of an appropriate grading order must be guided by a systematic, requirement-driven trade-off analysis.

## 4. Energy Harvesting Performance Analysis

Prior analytical investigations into vibration attenuation characteristics have demonstrated that appropriately engineered graded metamaterial structures—unlike conventional uniform configurations—exhibit superior broadband attenuation performance in the low-frequency regime. This enhanced behavior arises from the spatial frequency separation induced by bending wave propagation through the gradient beam, which in turn leads to localized energy concentration within the wavefield. Consequently, each segmented local oscillator embedded in the unit cell can efficiently capture vibrational energy at its intrinsic resonant band. Leveraging this wave manipulation principle, graded piezoelectric metamaterial beams hold significant promise for high-efficiency, low-frequency, and broadband vibrational energy harvesting.

Taking the power generation unit located in the *n*th segment of the *j*th unit cell as an example, the circuit governing equation for the piezoelectric element is derived by Kirchhoff’s current law [12,45]:

(22) $$C_{\left(\mathrm{ps},n\right)}v^{\prime }(t)+\frac{v(t)}{2Z}-\vartheta \left[\frac{\partial^{2}w_{n,j,2}(l_{2},t)}{\partial x\partial t}-\frac{\partial^{2}w_{n,j,2}(0,t)}{\partial x\partial t}\right]=0$$

where

(23) $$\vartheta =\frac{d_{31}b(h_{1}+h_{\left(2,n\right)})}{2s_{11}^{E}}$$

denotes the electromechanical coupling coefficient of the dual-functional system.

Following the Laplace transformation, the output voltage of the *n*th segment of the piezoelectric bimorph is derived as:

(24) $$V_{n,j}=\frac{2\vartheta s}{\left(1/Z+2sC_{\left(\mathrm{ps},n\right)}\right)}\left[\frac{\partial W_{n,j,2}(l_{2})}{\partial x}-\frac{\partial W_{n,j,2}(0)}{\partial x}\right]$$

Therefore, the output power of the piezoelectric bimorph can be expressed as:

(25) $$P_{n,j}=\frac{V_{n,j}^{2}}{2R}=\frac{{\left(\frac{2\vartheta s}{\left(1/Z+2sC_{\left(\mathrm{ps},n\right)}\right)}\left[\frac{\partial W_{n,j,2}(l_{2})}{\partial x}-\frac{\partial W_{n,j,2}(0)}{\partial x}\right]\right)}^{2}}{2R}$$

The output power *P_j_* of the *j*th unit cell is obtained by summing the output power contributions from all constituent segments.

(26) $$P_{j}=\sum_{n=1}^{S}\frac{{\left(\frac{2\vartheta s}{\left(1/Z+2sC_{\left(\mathrm{ps},n\right)}\right)}\left[\frac{\partial W_{n,j,2}(l_{2})}{\partial x}-\frac{\partial W_{n,j,2}(0)}{\partial x}\right]\right)}^{2}}{2R}$$

### 4.1. Analysis of Energy Harvesting Characteristics of a Unit Cell

**Figure 8:**
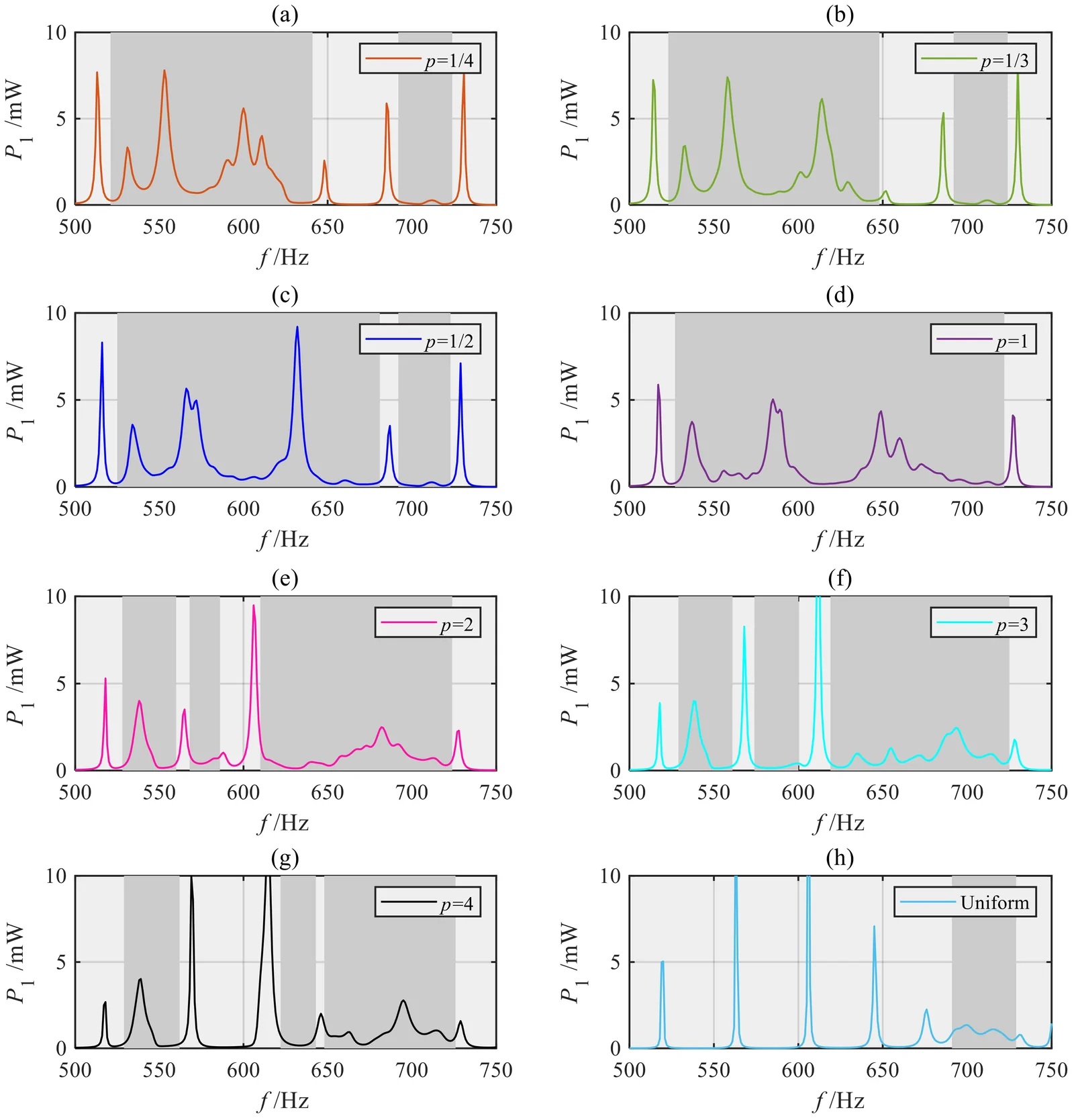
Frequency response curve of the output power for the first unit cell across various graded configurations.

This section numerically computes the output power of the first unit cell in graded-configured metamaterial beams, using Equation (26), and presents the corresponding frequency response curves of output power for each graded configuration, as illustrated in Figure 8. For comparative analysis, Figure 8 further includes the aggregate output power frequency response of the first ten oscillator units under the non-graded uniform configuration. Guided by the dual objective of simultaneous vibration attenuation and energy harvesting, this study specifically evaluates energy harvesting performance within the frequency bands where bending vibrations are effectively attenuated—bands previously identified through systematic vibration attenuation analysis. The shaded regions in Figure 8 denote these analytically validated bending vibration attenuation bands.

First, the energy harvesting characteristics of various graded configurations are systematically compared and analyzed within their respective vibration attenuation bands. Prior studies on vibration attenuation have demonstrated that fractional-order modes can induce a broad low-frequency bending vibration attenuation band. Within this attenuation band—often referred to as the “attenuation shadow region”—metamaterial-based graded structures exhibit continuous and pronounced broadband energy harvesting performance, as illustrated in Figure 8a–c. As the grading exponent *p* increases, the effective energy harvesting bandwidth progressively expands, attaining its maximum when *p* approaches unity. Figure 8e–g present the frequency response curves of output power associated with higher-order modes; these curves confirm that such modes also support broadband energy harvesting, albeit with a significantly narrower effective bandwidth and a spectral shift toward higher frequencies relative to the first-order mode. In contrast, the non-graded configuration (Figure 8h) exhibits only limited energy harvesting capability within the attenuation region, owing to its inherently narrow vibration attenuation bandwidth.

To quantitatively assess the variations in energy harvesting performance across various graded configurations, this study adopts two objective metrics: the energy harvesting bandwidth Δ*f*—which is equivalently defined as the vibration attenuation bandwidth—and the energy harvesting area Ψ. The corresponding computational formulations are provided in Equation (27).

(27) $$\Psi =\sum_{i=1}^{t}\int_{f_{si}}^{f_{ci}}P_{j}(f)\mathrm{df}t=1,2,3\cdots$$

**Figure 9:**
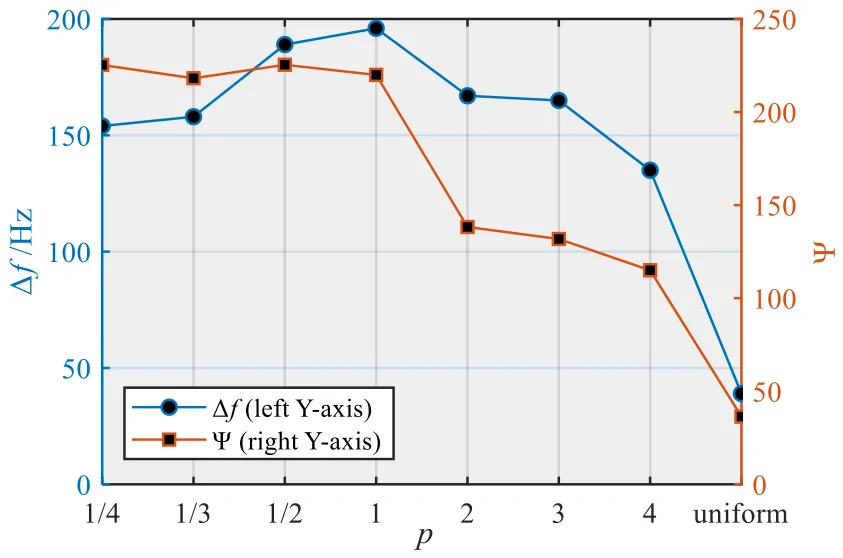
Variation of energy harvesting area and bandwidth across various graded configurations.

The energy harvesting areas corresponding to each graded configuration are computed using Equation (27); the results are presented in Figure 9. As shown, the trend in the energy harvesting area closely parallels that of the vibration attenuation area: configurations associated with fractional-order and first-order modes exhibit substantially larger energy harvesting areas, whereas higher-order modes demonstrate progressively diminished performance. A pronounced stepwise reduction occurs between the first and second orders, followed by a gradual, monotonic decline with increasing grading order. For comparative purposes, Figure 9 also includes the energy harvesting area for the non-graded configuration, which—owing to its narrow vibration attenuation band—yields comparatively low energy harvesting efficiency. In contrast, graded configurations facilitate spatially distributed bandgap formation across unit cell segments, each tuned to resonate at distinct frequencies; this enables targeted energy harvesting within multiple, non-overlapping bandgap ranges. This multi-band, cooperative energy harvesting mechanism markedly improves overall harvesting performance—particularly for fractional-order, first-order, and selected higher-order graded configurations—relative to conventional non-graded designs.

### 4.2. Analysis of Energy Harvesting Characteristics for Each Segment Within a Unit Cell

**Figure 10:**
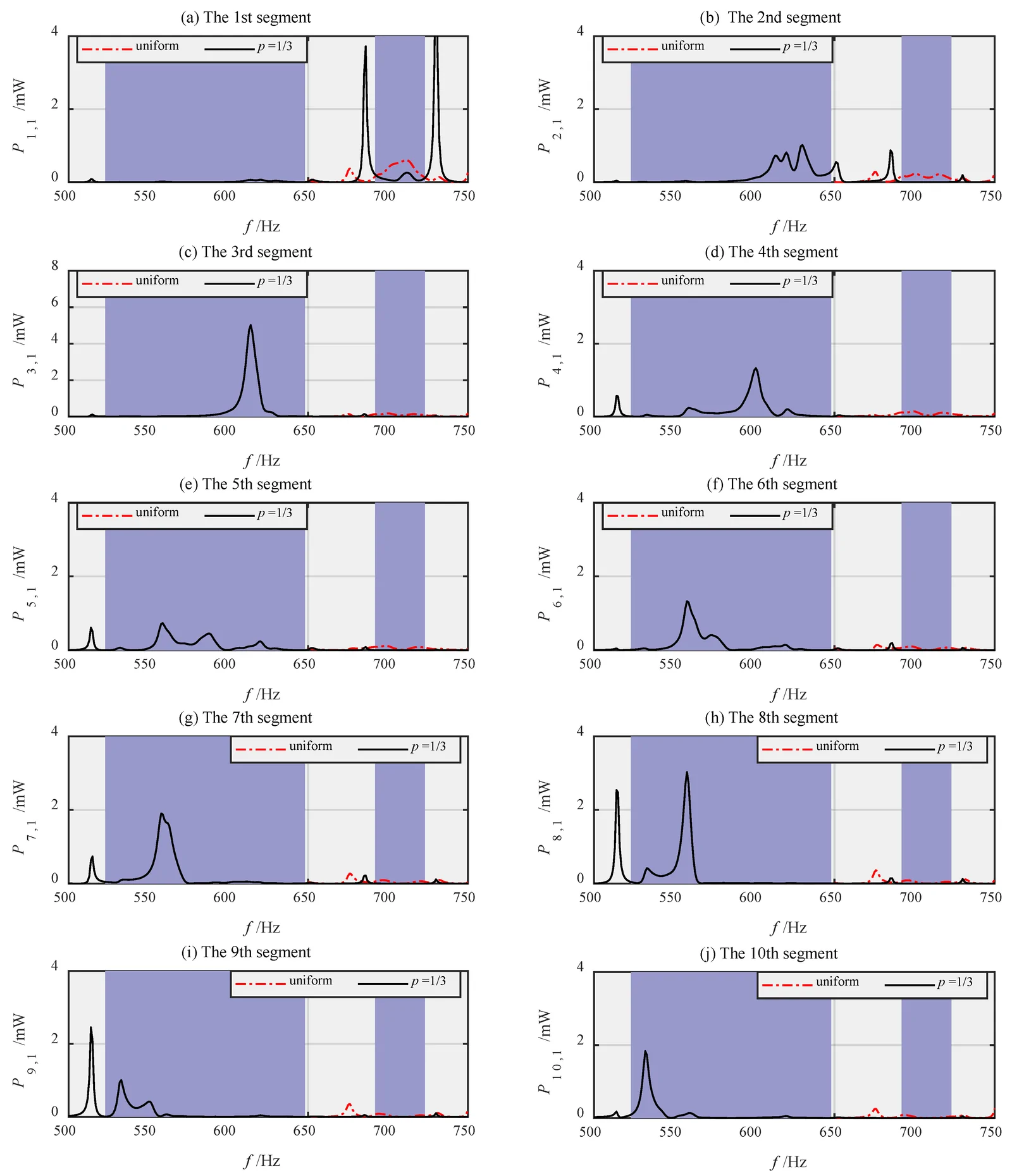
Frequency response curves of the output power for individual segments within the first unit cell of graded and non-graded metamaterial beams. (The frequency range calculated for the non-graded configuration is 650–750 Hz).

To elucidate the energy harvesting behavior of segmented oscillators within graded metamaterial unit cells, this section compares a fractional-order graded configuration (*p* = 1/3) with a non-graded counterpart. Power output distributions across individual oscillators are systematically analyzed. Numerical calculation results are presented in Figure 10, wherein shaded regions denote the vibration attenuation bands for the 1/3-order graded configuration. For clarity and to highlight the effective frequency range of both graded and non-graded configurations, only data proximal to the vibration attenuation band (650–750 Hz) are displayed for the non-graded case, revealing the variation of segmented energy harvesting frequency bands under graded configurations.

As observed, energy harvesting in the non-graded structure is predominantly localized near the excitation end—a phenomenon consistent with prior findings and therefore not reiterated herein. In contrast, the graded configuration enables efficient, segment-specific energy harvesting across distinct resonant frequency intervals. The corresponding energy harvesting bands for segments 1 through 10 are: (707–717 Hz), (600–648 Hz), (594–629 Hz), (557–611 Hz), (553–594 Hz), (548–581 Hz), (540–572 Hz), (531–564 Hz), (528–554 Hz), and (524–544 Hz). Each segment exhibits an independent yet partially overlapping harvesting band; notably, the 10th segment operates at the lowest frequency range, while successive segments toward the excitation end shift progressively toward higher frequencies—thereby manifesting a well-defined spatial-frequency gradient in wave energy localization. Owing to inter-segment band overlap and coupling (except for segment 1), the overall structure achieves continuous broadband energy harvesting capability. The aggregate output power spectrum is shown in Figure 8b.

In summary, introducing the gradient concept significantly broadens the operational bandwidth of the dual-functional piezoelectric metamaterial beam. Compared to the non-graded uniform configuration, under identical conditions, the proposed gradient design achieves improvements of approximately 402.6% in attenuation bandwidth *(p* = 1), 47% in attenuation area (*p* = 1/4), and 519.9% in energy harvesting area (*p* = 1/2). This effectively addresses the relatively narrow dual-functional bandwidth limitation commonly observed in conventional piezoelectric metamaterial beam structures [38,39,42,43,44], substantially enhancing their dual-functional performance. Moreover, in contrast to existing studies on graded piezoelectric metamaterial dual-function beams, the proposed architecture achieves axial-gradient modulation of the geometric symmetry of local resonators solely through structural tailoring—without requiring adjustments to external circuit parameters. This eliminates a key limitation of prior approaches [40,41], which relied on electrical parameter tuning to realize graded functionality. Furthermore, unlike our earlier non-periodic implementation, the present design enforces strict periodicity across unit cells, enabling rigorous band structure analysis and direct characterization of bandgap properties—thus resolving a fundamental theoretical constraint in previous studies [45].

## 5. Finite Element Simulation Analysis

To validate the theoretical predictions regarding the dual-functional characteristics of graded piezoelectric metamaterial beams, this section presents finite element simulations performed using COMSOL Multiphysics 5.4—a commercially available, high-fidelity multiphysics simulation platform. All key modeling parameters—including geometric configuration, constituent material properties, piezoelectric coefficients, and electromechanical boundary conditions—are rigorously aligned with those adopted in the preceding theoretical analysis.

The finite element simulation parameter settings are shown in Table 2:

### 5.1. Simulation Analysis of Vibration Attenuation Performance

**Figure 11:**
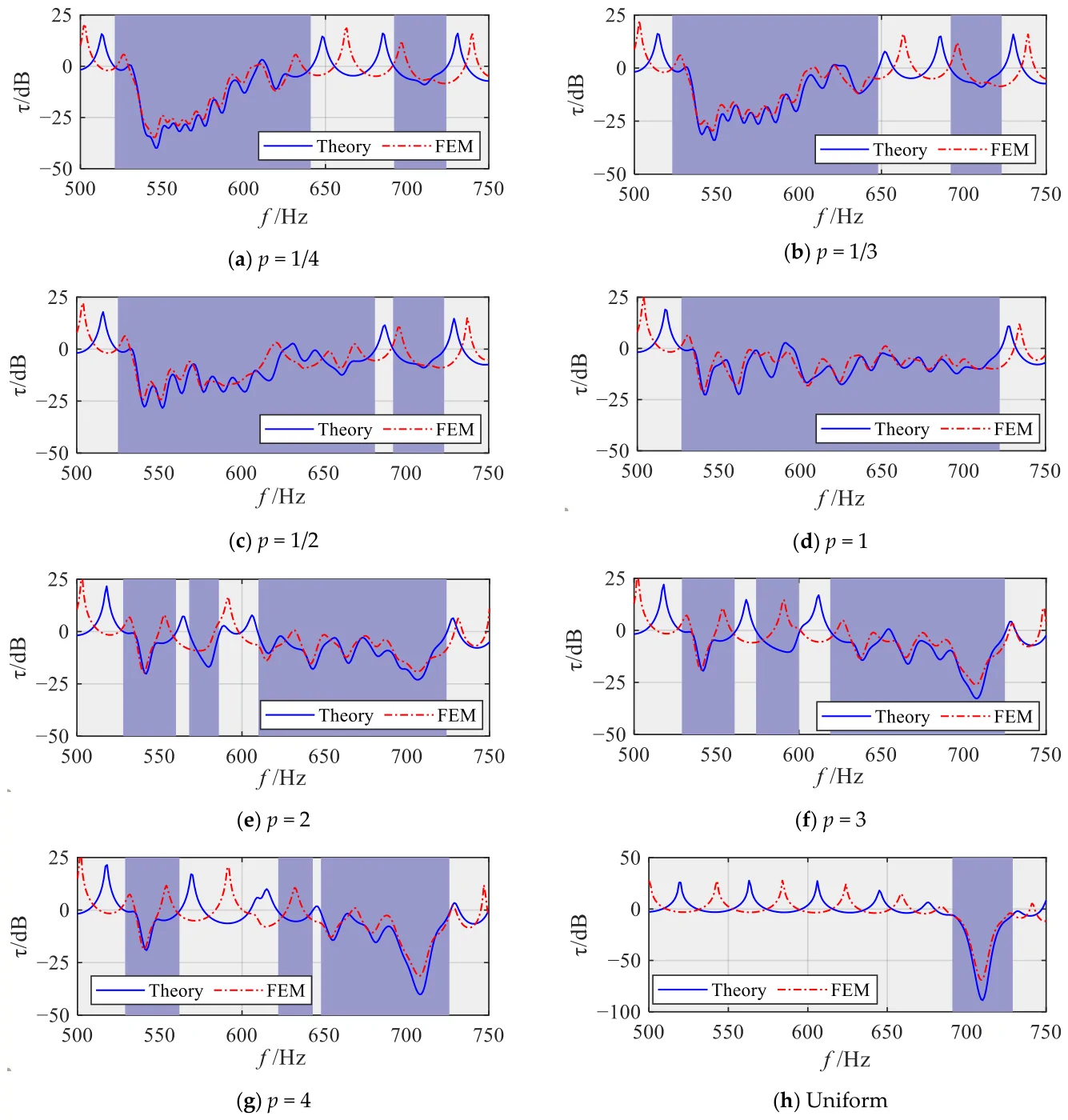
Comparison of simulated and theoretical vibration transmission characteristics across various graded configurations.

First, the structural vibration attenuation characteristics are validated via numerical simulation. Figure 11 presents a comparative analysis of the theoretical and finite element simulated bending vibration transmissibility frequency response curves across various graded orders (*p*). The shaded regions denote the theoretically predicted vibration attenuation bands associated with each graded configuration. Within these bands, the finite element simulated curves consistently exhibit measurable reductions in vibration amplitude—demonstrating qualitative agreement with theoretical predictions. Specifically, fractional-order graded configurations yield dominant attenuation in the low-frequency regime, whereas higher-order configurations extend effective attenuation into the mid- to high-frequency ranges. Overall, the theoretical and finite element curves align closely in terms of global trend and band structure; minor discrepancies in peak and valley magnitudes within the attenuation bands are attributed to modeling approximations rather than fundamental disagreement. The structural gradient characteristics impose inherent constraints on pointwise relative error computation across the entire frequency band. To enable a meaningful and representative comparison, three characteristic frequencies are selected: the valleys nearest to the upper and lower boundaries of the attenuation band and the frequency corresponding to maximum attenuation. As summarized in Table 3, both theoretical predictions and simulation results are provided for these frequencies, along with the corresponding relative errors. All computed relative errors remain below 1%, demonstrating excellent quantitative agreement between the analytical model and numerical simulation.

**Figure 12:**
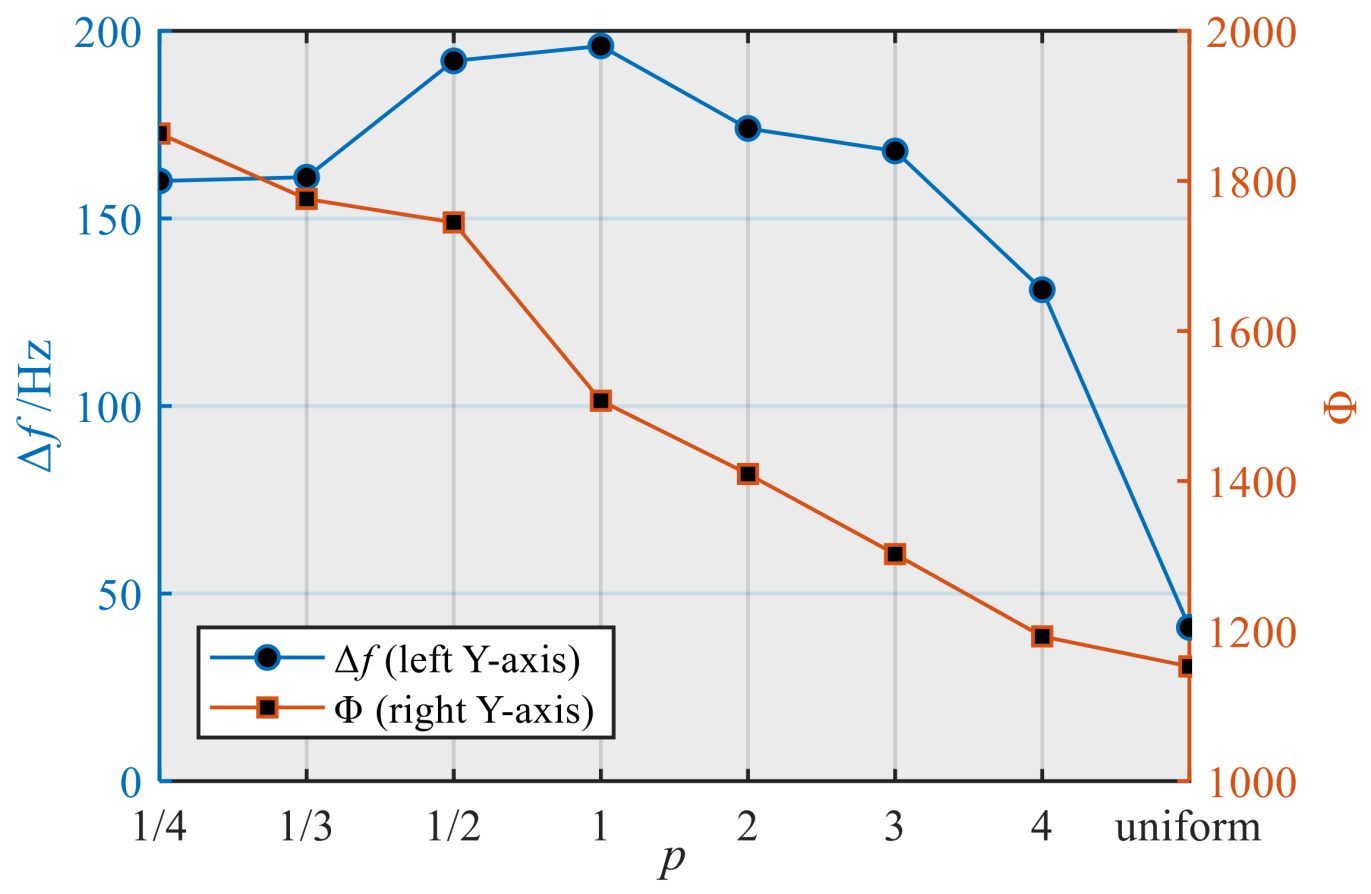
Simulation results of variation of attenuation bandwidth and attenuation area across various graded configurations.

Furthermore, to comprehensively verify the attenuation characteristics of the metamaterial beam across the entire attenuation frequency band, this study further analyzes the attenuation bandwidth (Δ*f*) and attenuation area (Φ) under various graded orders. Based on the simulated frequency response curves of transmission rate shown in Figure 11, the variation trends of attenuation bandwidth and attenuation area for various graded orders are plotted in Figure 12. The results indicate that as the graded order *p* increases, the attenuation bandwidth initially increases and then decreases, with the first-order gradient configuration exhibiting the maximum global attenuation bandwidth, while the uniform structure has the smallest attenuation bandwidth. Meanwhile, the attenuation area generally decreases with increasing graded order, where fractional-order configurations show the largest attenuation area, followed by integer-order configurations, and the uniform structure exhibits the smallest attenuation area. These findings are consistent with the theoretical analysis presented in Figure 7.

Outside the shaded attenuation bands—i.e., in non-attenuating frequency regions—the divergence between the two curves becomes more pronounced, primarily due to shifts in resonance peak frequencies. This frequency shift stems from inherent differences in modeling assumptions: the theoretical model employs the classical Euler–Bernoulli beam theory, which neglects shear deformation and rotary inertia effects; in contrast, the finite element model incorporates both effects comprehensively. As shear deformation reduces the effective bending stiffness and rotary inertia increases the effective mass moment of inertia, the resulting natural frequencies in the finite element model are systematically lower than those predicted by the simplified theoretical formulation.

### 5.2. Simulation Analysis of Energy Harvesting Performance

This section presents a comprehensive validation and analysis of the energy harvesting performance of graded piezoelectric metamaterial beams via finite element simulation. The investigation centers on two complementary aspects: (i) the macroscopic energy harvesting characteristics of the unit cell and (ii) the spatially resolved energy harvesting behavior across individual segments within the unit cell.

**Figure 13:**
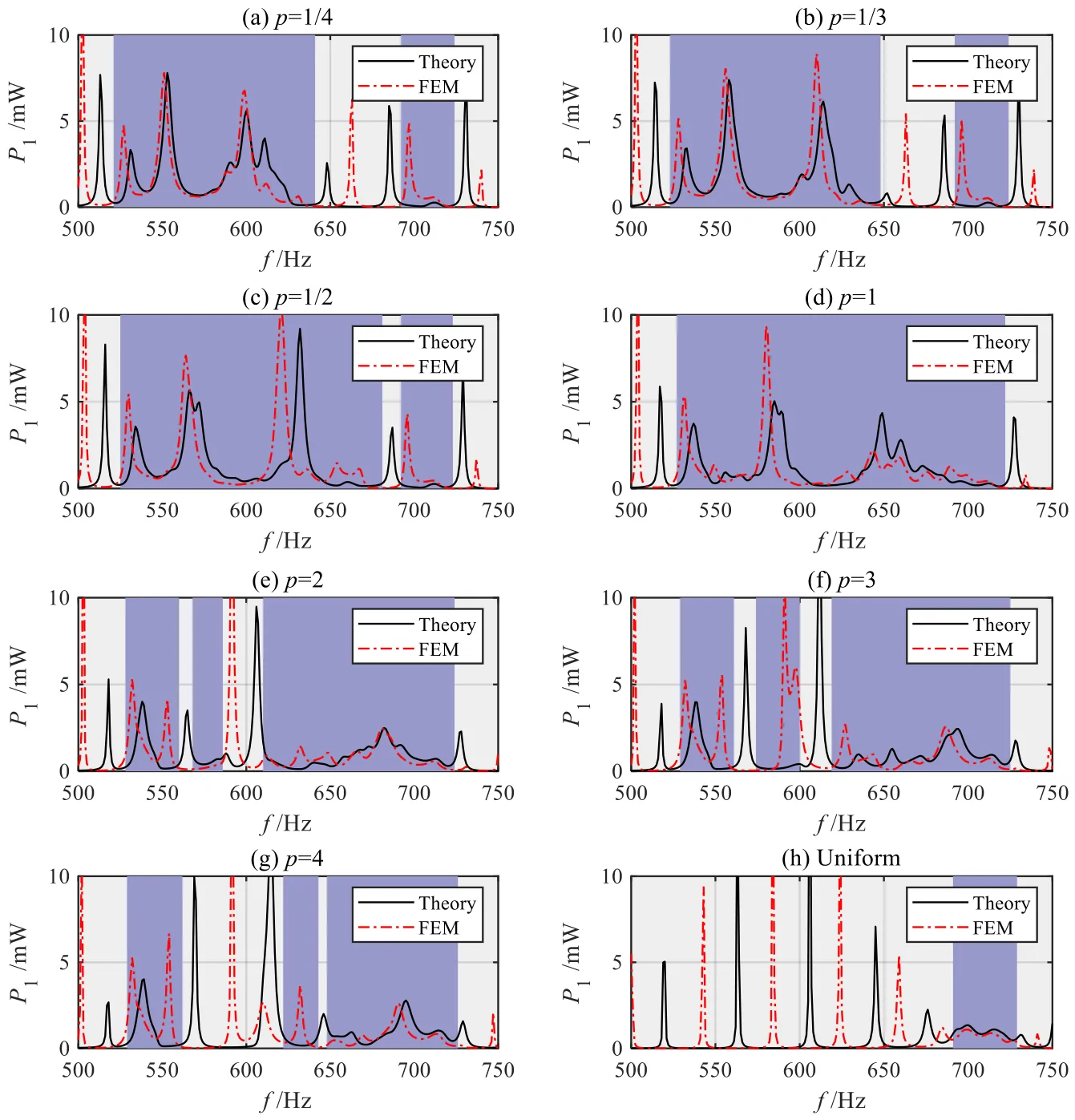
Simulation and theoretical frequency response curves of the output power for the first unit cell across various graded configurations.

Initially, the output power generated by the unit cell is quantified through simulation. Figure 13 presents a comparative analysis of the simulated and theoretical frequency response curves of the output power for metamaterial unit cells under various graded configurations. The shaded regions denote the theoretically predicted attenuation bands associated with each graded design. All graded configurations demonstrate pronounced broadband energy harvesting capability within their respective theoretical attenuation bands. Within these effective attenuation bands, the simulated and theoretical output power curves exhibit strong qualitative agreement in overall trend; however, minor discrepancies are observed in both the locations and amplitudes of specific power peaks. Notably, the simulated peak frequencies consistently occur at slightly lower values than those predicted by theory. Analogous to the simulation analysis of vibration attenuation performance, Table 4 lists the theoretical values, simulation results, and corresponding relative errors for the three characteristic frequencies—namely, those associated with the peak and valley points of the output power curve within the shaded frequency region—for each grading mode. The results indicate that the relative errors at these characteristic frequencies are all less than 2%, demonstrating good agreement between the theoretical predictions and the simulation results. This systematic downward shift arises from the intrinsic bandgap characteristics of the local oscillators embedded in each segment of the graded unit cell. Due to coupling and partial overlap between adjacent resonant bandgaps, localized modulations emerge within the attenuation band of the finite-periodic metamaterial beam—these modulations directly correspond to the observed power peaks in the energy harvesting response. As corroborated by prior vibration attenuation simulations, the finite element model rigorously incorporates shear deformation and rotary inertia effects—factors neglected in the classical Euler-Bernoulli beam formulation—thereby yielding a lower global natural frequency spectrum and accounting for the consistent low-frequency shift in harvested power peaks. Compared to the attenuation bands, the discrepancies between the simulated and theoretical curves are more pronounced in the non-attenuation regions, where the simulated peak frequencies again occur at lower values—a trend consistent with the underlying modeling assumptions.

**Figure 14:**
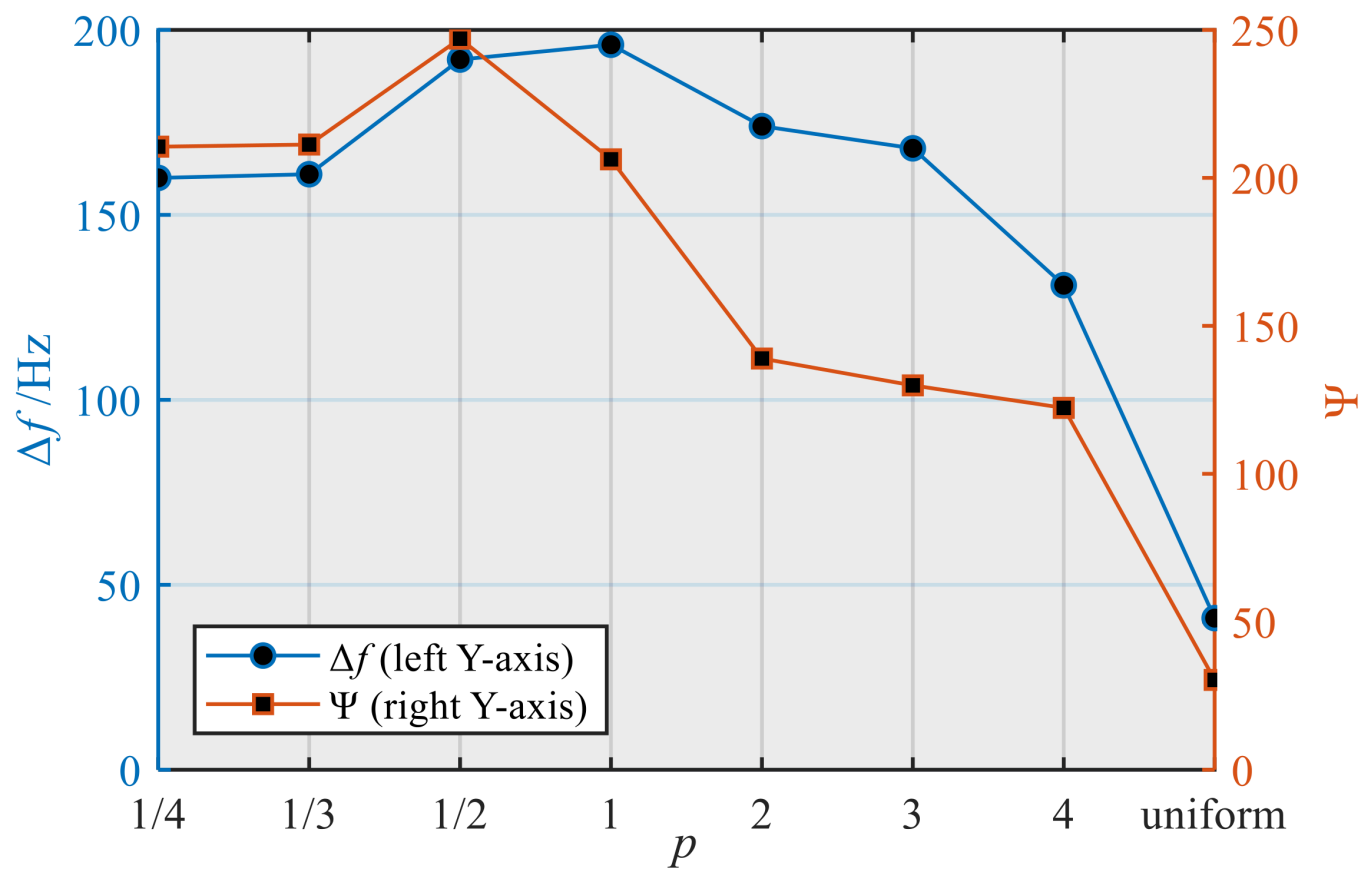
Simulation results of variation of energy harvesting area and bandwidth across various graded configurations.

Furthermore, to comprehensively verify the energy harvesting characteristics of the structure across the entire dual-functional frequency band, this study further analyzes the energy harvesting area (Ψ) of the metamaterial beam under various graded configurations. Based on the simulated attenuation band in Figure 11 and the energy harvesting frequency response curve shown in Figure 13, the variation curves of the energy harvesting area for various graded orders are plotted in Figure 14. The results indicate that the energy harvesting areas of fractional-order and first-order configurations are significantly larger, while higher-order configurations exhibit relatively lower overall energy harvesting performance. A distinct stepwise reduction in the energy harvesting area occurs between the first and second orders, followed by a continuous decline as the grading order increases. Additionally, the non-graded configuration has a narrower vibration attenuation band, resulting in a low energy harvesting area—the smallest among all configurations. This trend is consistent with the theoretical analysis conclusions presented in Figure 9.

**Figure 15:**
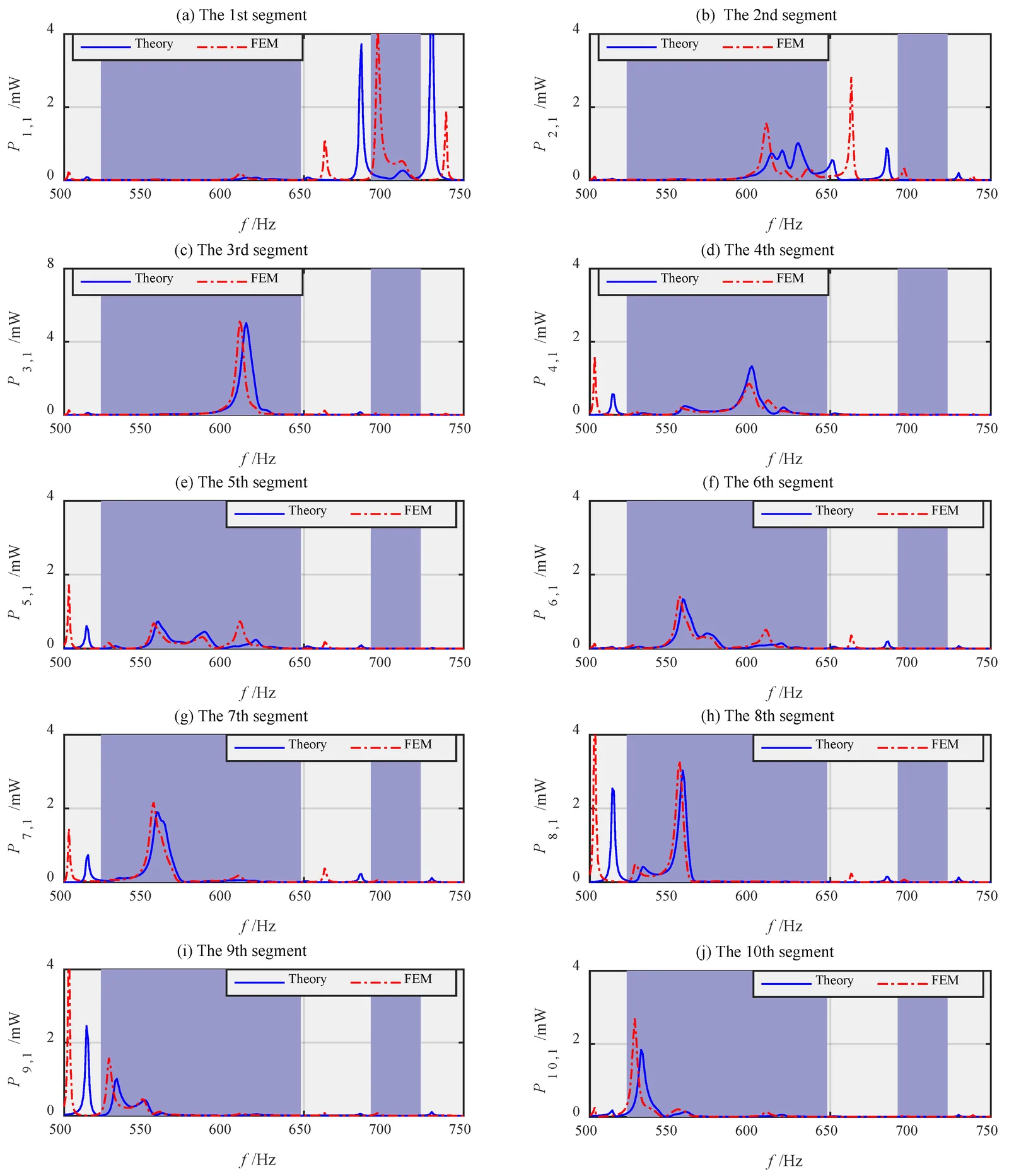
Simulation and theoretical frequency response curves of the output power for individual segments within the first unit cell of a graded piezoelectric metamaterial beam (*p* = 1/3 order).

To rigorously validate the energy harvesting mechanisms associated with segmented oscillators in a graded metamaterial beam unit cell, this study selects the 1/3-order graded configuration as a representative case and performs finite element simulations to characterize the energy harvesting performance of each oscillator segment. Figure 15 compares the simulated and theoretically predicted output power responses. Meanwhile, Table 5 summarizes the theoretical values, simulation results, and relative errors of the characteristic frequencies corresponding to the peak points of the output power curves within the shaded frequency region. The results indicate that the relative errors at the characteristic frequency positions are all less than 1%. Furthermore, the simulated frequency response curves align closely with theoretical predictions across all segments. Specifically, each oscillator achieves efficient energy harvesting within its designated resonant band; moreover, the effective harvesting bands exhibit a systematic downward shift—from mid-to-high frequencies in upstream segments to lower frequencies in downstream segments—consistent with the imposed graded design. The intentional spectral overlap and inter-segment coupling collectively enable broadband energy harvesting capability, as evidenced by the smooth and broadened total output power spectrum in Figure 13b. Overall, the simulation results demonstrate strong quantitative agreement with analytical theory. Only minor discrepancies exist in peak power amplitudes and resonant frequency positions, which arise from modeling differences between the simplified theoretical model and the refined finite element model. Crucially, these discrepancies remain within acceptable bounds and do not compromise the physical validity or conceptual soundness of the proposed energy harvesting mechanism.

The theoretical model adopted in this study is based on the Euler–Bernoulli beam assumption, which neglects shear deformation and rotary inertia. This simplification is well-established for periodic metamaterial beams with a high thickness-to-length ratio and aligns consistently with our research group’s prior theoretical studies on analogous structures—thereby enabling systematic, cross-study comparisons. Within the target operational frequency band—specifically, the band where vibration attenuation and mechanical energy harvesting coexist—the theoretical predictions exhibit strong agreement with finite element simulations, except for slight deviations in the positions and amplitudes of a few peaks and valleys. These deviations arise jointly from the inherent assumptions of the Euler–Bernoulli formulation and numerical artifacts intrinsic to multi-physics finite element modeling (e.g., mesh discretization, coupling scheme, and boundary condition implementation). Crucially, despite these quantitative discrepancies, the model robustly captures the essential wave propagation characteristics, the underlying energy localization and conversion mechanisms of bending elastic waves, and the parametric trends governing dual-function performance across graded configurations. As such, it substantiates the study’s central conclusion. For future studies involving thicker beams, the Timoshenko beam theory—which accounts for shear deformation and rotary inertia—may be adopted to improve predictive accuracy.

## 6. Conclusions

This study employs graded piezoelectric metamaterial beams as a model platform to systematically investigate the underlying mechanisms governing bending wave manipulation, as well as the dual functionality of simultaneous vibration attenuation and energy harvesting. Through integrated theoretical modeling, mechanistic analysis, parametric pattern exploration, and finite element simulation validation, the study comprehensively elucidates how gradient structural configurations modulate wave propagation behavior and enable synergistic dual performance. These findings directly overcome key limitations inherent in conventional uniform piezoelectric metamaterials—particularly their narrow operational bandwidth and suboptimal low-frequency response. The principal research conclusions are summarized as follows:

(1) A grading-based piezoelectric metamaterial beam analytical model was developed to enable precise, controllable design of the resonant frequency gradient for localized oscillators. The thickness profile of the piezoelectric layer exhibits distinct spatial distribution characteristics governed by the grading order: fractional-order grading yields a front-to-back sparsity-to-density transition; first-order grading produces a uniform, linearly varying thickness distribution; and higher-order grading induces a front-to-back density-to-sparsity transition. This engineered graded architecture constitutes a fundamental enabler for spatial frequency separation of bending waves and the realization of broadband bandgap characteristics.

(2) The underlying mechanisms governing wave manipulation and energy localization in graded metamaterials have been elucidated. The spatially varying graded configuration facilitates frequency-dependent separation of bending waves and induces progressive energy localization across unit cell segments. Specifically, bending waves of distinct frequencies sequentially attain zero group velocity and become spatially confined within designated segments. Consequently, localized oscillators embedded in each segment operate efficiently within their respective resonance bands, enabling concurrent vibration attenuation and energy harvesting—thereby realizing functionally integrated, synergistic performance.

(3) The regulatory mechanism by which grading order distribution governs vibration attenuation performance has been elucidated. Specifically, bending-wave bandgaps associated with fractional-order graded configurations are predominantly localized in the low-frequency regime, thereby delivering superior low-frequency vibration attenuation. With increasing grading order, the attenuation bandwidth first broadens and subsequently narrows, reaching its maximum for the first-order configuration. Higher-order graded configurations progressively shift the effective attenuation band toward higher frequencies while concurrently reducing its width. Crucially, compared to non-graded uniform configurations, the proposed graded design markedly extends the overall frequency range over which effective vibration attenuation is achieved—thereby mitigating the inherent narrowband limitation characteristic of conventional passive attenuation structures.

(4) This study elucidates the broadband energy harvesting mechanisms and quantitatively compares the performance characteristics across distinct graded configurations. The progressively varying structural design allows each segmented oscillator to exhibit continuously tunable resonant frequencies; consequently, the energy harvesting bands of adjacent segments overlap and couple, collectively yielding a continuous broadband harvesting response. Quantitative analysis reveals that fractional-order and first-order graded configurations achieve the optimal energy harvesting bandwidth and coverage area. In contrast, higher-order graded configurations shift their dominant harvesting bands toward higher frequencies while exhibiting reduced bandwidth. The non-graded uniform configuration demonstrates the lowest overall energy harvesting efficiency and narrowest operational bandwidth.

Although the present study has yielded the aforementioned theoretical advances and conceptual innovations, several limitations warrant acknowledgment. First, current research on dual-function characteristics predominantly addresses forward problem analysis—that is, characterizing dual-function behavior given fixed model parameters—whereas efficient inverse design methodologies capable of synthesizing structures to achieve prescribed dual-function performance hold significantly greater practical relevance for engineering implementation. Second, experimental verification remains absent in this work. As a preliminary theoretical investigation, the primary contribution of this study lies in proposing a novel theoretical model, demonstrating the feasibility and potential value of broadband dual-function characteristics from a theoretical perspective, and establishing a foundation for subsequent experimental efforts. Regarding theory–experiment alignment, the proposed model reliably captures qualitative trends in dual-function response curves across varying graded configurations and exhibits strong agreement with anticipated experimental observations. However, quantitative discrepancies between theoretical predictions and empirical measurements may arise due to deliberate model simplifications—including the omission of shear deformation, rotary inertia, and structural damping. Consequently, while the model does not substitute for experimental validation, it offers robust physical insight and actionable qualitative guidance for future experimental investigations.

## Figures and Tables

**Table 1:** Geometric, material, and circuit parameters of the graded metamaterial beam.

Items	Parameters	Values	Units
Substrate (PMMA)	*ρ*_1_	1142	kg/m^3^
	*E*_1_	2.0	GPa
	*b*	20	mm
	*h*_1_	5	mm
	*l*	65	mm
	*l*_1_	10	mm
Piezoelectric bimorph (PZT-5H)	$s_{11}^{E}$	1.65 × 10^−11^	m^2^/N
	$\varepsilon_{33}^{T}$	3.01 × 10^−8^	F/m
	*d*_31_	−2.74 × 10^−10^	C/N
	*ρ*_2_	7500	kg/m^3^
	*l*_2_	45	mm
	*h*_2_	0.35	mm
	Δ*h*_2_	0.15	mm
Circuit	*L*	330	mH
	*R*	100	kΩ
Periods	*j*	3	
Segments	*S*	10	

**Table 2:** Environment settings of finite element simulation analysis.

Item	Setting
Gradient mode	1/4, 1/3, 1/2, 1, 2, 3, 4, uniform
Spatial dimension	2D
Physical field	Solid Mechanics-Piezoelectric Devices-Circuits
Meshing	Free triangular mesh
Mesh density	Extra fine
Boundary conditions	An acceleration amplitude of 10 g is applied on the left end face, and the right end face is free
Research type	Frequency domain analysis
Frequency sweep range	500–750 Hz
Step size	1 Hz
Polarization direction	Vertical upward
Piezoelectric bimorph connection type	Parallel connection
Circuits	*L*-*R* parallel connection

**Table 3:** Comparison of simulation and theoretical results for the characteristic frequencies of the vibration transmission curves across various graded configurations.

*p*	1/4	1/3	1/2	1	2	3	4	Uniform
Theory (Hz)	547	541	541	542	542	542	542	710
	547	548	552	562	706	708	708	
	620	636	661	696	706	708	708	
FEM (Hz)	545	540	540	540	540	541	541	709
	545	547	551	561	706	707	708	
	620	636	660	695	706	707	708	
Relative errors (%)	0.37	0.18	0.18	0.37	0.37	0.18	0.18	0.14
	0.37	0.18	0.18	0.18	0	0.14	0	
	0	0	0.15	0.14	0	0.14	0	

**Table 4:** Comparison of simulation and theoretical results for the characteristic frequencies of the output power frequency response curves across various graded configurations.

*p*	1/4	1/3	1/2	1	2	3	4	Uniform
Theory (Hz)	531	533	534	537	538	538	539	700
	553	558	566	585	641	635	695	708
	600	614	632	649	682	694	714	715
FEM (Hz)	527	528	530	531	532	532	532	699
	551	556	564	580	632	627	691	707
	599	610	621	644	681	687	713	714
Relative errors (%)	0.75	0.94	0.75	1.12	1.12	1.12	1.30	0.14
	0.36	0.36	0.35	0.85	1.40	1.26	0.58	0.14
	0.17	0.65	1.74	0.77	0.15	1.01	0.14	0.14

**Table 5:** Comparison of simulation and theoretical results for the characteristic frequencies of the output power frequency response curves in each segment of a unit cell.

*n*	1	2	3	4	5	6	7	8	9	10
Theory (Hz)	712	613	614	601	559	558	558	558	533	532
FEM (Hz)	711	610	610	599	556	556	556	556	528	528
Relative errors (%)	0.14	0.49	0.65	0.33	0.54	0.36	0.36	0.36	0.94	0.75

## Data Availability

The original contributions presented in this study are included in the article. Further inquiries can be directed to the corresponding author.
